# Epidemiology and Clinical Features of Mpox in Jakarta, Indonesia, August 2022–December 2023

**DOI:** 10.3390/vaccines13030210

**Published:** 2025-02-20

**Authors:** Hanny Nilasari, Eliza Miranda, Melani Marissa, Ani Ruspitawati, Dwi O. T. L. Handayani, Ngabila Salama, Budi Setiawan, Supriadi, Tiranti V. Aisyah, Inggariwati, Arif S. Haq, Siti Zuhroh, Eka Y. Safitri, Rahmat A. Pramono, Inggrita Wisnuwardani, Erni J. Nelwan, Robert Sinto, Adityo Susilo, Yulia R. Saharman, Suratno L. Ratnoglik, Ni L. P. Pitawati, Muhammad Fauzan, Sekar S. A. Hasanah, Megandhita Sharasti, Evy Yunihastuti

**Affiliations:** 1 Cipto Mangunkusumo Hospital, Jakarta 10430, Indonesia; mirandaeliza74@gmail.com (E.M.); melani_marissa@yahoo.com (M.M.); erni.juwita@ui.ac.id (E.J.N.); robert.sinto01@ui.ac.id (R.S.); adityo_susilo@yahoo.com (A.S.); yulia.rosa18@gmail.com (Y.R.S.); sl.ratnoglik@yahoo.com (S.L.R.); evy.yunihastuti@gmail.com (E.Y.); 2 Department of Dermatology and Venereology, University of Indonesia, Jakarta 10430, Indonesia; 3 DKI Jakarta Health District, Jakarta 10160, Indonesia; dinkes@jakarta.go.id (A.R.); dokterlies@gmail.com (D.O.T.L.H.); ngabilas@gmail.com (N.S.); surveilansdinkesdki2021@gmail.com (B.S.); supriadihadimulyono.skm@gmail.com (S.); vindha.aisyah@gmail.com (T.V.A.); inggariwati@gmail.com (I.); arifsyaifulhaq@gmail.com (A.S.H.); zuhroh_280186@yahoo.com (S.Z.); alliesafitri@gmail.com (E.Y.S.); p2pdinkesdkijakarta@gmail.com (R.A.P.); imunisasidinkesdki@gmail.com (I.W.); 4 Department of Internal Medicine, University of Indonesia, Jakarta 10430, Indonesia; 5 Department of Clinical Microbiology, University of Indonesia, Jakarta 10430, Indonesia; 6 National Infectious Disease Center Sulianti Saroso Hospital, Jakarta 14340, Indonesia; ppitawati@yahoo.com (N.L.P.P.); dr.fauzan90@gmail.com (M.F.); 7 Faculty of Medicine, Trisakti University, Jakarta 11440, Indonesia; sekar.saras17@gmail.com; 8 Faculty of Medicine, University of Indonesia, Jakarta 10430, Indonesia; mail.sharasti@gmail.com

**Keywords:** epidemiology, clinical features, mpox, HIV

## Abstract

Background/Objective: This study explores the epidemiology and clinical features of re-emerging mpox in Jakarta, Indonesia. Methods: This study used a retrospective study design to describe the epidemiological data, clinical features, and mortality of mpox patients from August 2022 to December 2023. In addition, this study also aims to identify the differences in both the epidemiology and clinical features of mpox in people living with HIV (PLHIV) and in non-HIV patients (non-PLHIV). Results: Our study shows that, as of the end of December 2023, 59 mpox cases were treated in Jakarta. All of the mpox cases in Jakarta were diagnosed in males, mainly found in MSM (91.5%), and PLHIV (78%). Most patients would manifest with fever, rash, and skin lesions. Syphilis was found as a concomitant infection in this group (22/59, 37.2%). Severe manifestations were found among PLHIV without antiretroviral therapy (ART). Conclusions: Mpox cases in Jakarta were all found in males and most of them were PLHIV. There are various manifestations of mpox; however, since immunosuppressed patients could present differently, a strong surveillance and vaccine notification system, cautious management, and spreading vaccination awareness are needed to prevent and treat mpox.

## 1. Introduction

Mpox is an emerging zoonotic disease caused by the monkeypox virus (MPXV), which belongs to the Poxviridae family and the Orthopoxvirus genus. Mpox was first detected in 1958 in Denmark when two cases similar to smallpox appeared in monkeys under study, hence the name ‘monkeypox’ or mpox [1,2]. MPXV came from the same family as the smallpox-causing virus, and smallpox was successfully eradicated globally in 1980. There is a vaccination for smallpox and it also gives cross-immunity to MPXV. However, despite the success in eliminating smallpox, mpox continues to spread sporadically and has become endemic in certain regions of Africa, particularly in Central and West Africa, where it persists as a public health challenge [3,4]. Around July 2022, a multi-country outbreak of mpox happened and slowly extended to parts of Asia, with approximately 100 case notifications for mpox from South-East Asia in August 2023 [5,6]. Indonesia reported 88 confirmed cases in September 2024, underscoring the growing presence of this re-emerging disease in the region, and Jakarta has the highest number of cases [7]. MPXV is characterized by double-stranded DNA and divided into two taxonomic groups, which are Clade I and Clade II; each consists of two subclades (Clade Ia and Ib, Clade IIa and IIb) [8]. Clade I has a mortality rate of up to 10%, while Clade II has a mortality rate of less than 1%. The MPXV strain involved in the current outbreak is classified as Clade IIb [9]. Based on the Indonesian report, 54 cases out of 88 cases in Indonesia met the criteria for whole genome sequencing (WGS), and the results of WGS showed that all 54 cases were caused by the Clade IIb strain [10]. In response to the first reported case in 2022, Indonesia implemented a specialized epidemiological investigation form as part of its efforts to monitor and address the ongoing threat posed by mpox.

Monkeypox virus can be transmitted from human to human by droplets, respiratory secretions, intimate contact, skin lesions, and contaminated objects [11]. Men who have sex with men (MSM) and bisexuals are at increased risk of MPXV infection, which indicates that mpox is more likely to spread among people within the close-contact community [12]. Human-to-human transmission occurred in the mpox outbreak in 2022 [9]. Most of the mpox cases are symptomatic. However, about 53% of transmission might occur in the pre-symptomatic phase, and asymptomatic cases could also transmit the infection; thus, contact tracing is needed to contain the outbreak [13,14].

According to the Indonesian guideline for the prevention and control of mpox by the Indonesian Health Ministry, the severity index could correlate with the prognosis of the case. The criteria of the severity index are whether the patient is in one of the following high-risk groups: children under eight years old, pregnant women, immunocompromised patients, and patients with chronic skin conditions; having one sign of complication symptoms; with three abnormal laboratory examinations, including whether it is aspartate aminotransferase or alanine transaminase increase, leukocytosis, decreasing blood urea nitrogen, thrombocyte, or albumin; and a severe or very severe skin-lesion amount. The case is considered a mild degree case if they did not meet the criteria mentioned above. It would be regarded as a severe degree case if they fulfilled at least one of the criteria [3].

Although cases may recover well, a study found that 30.18% of the confirmed cases were HIV positive, which poses a higher risk of mortality, especially when the patient is immunocompromised [15,16]. Therefore, more attention would be needed for mpox patients with HIV.

One of the recommended prevention strategies against MPXV infection is vaccination. Modified vaccinia Ankara (MVA) vaccine, Bavarian Nordic (JYNNEOS), and ACAM2000 are currently two available vaccines that are already approved by the Food and Drug Administration (FDA) to prevent mpox and smallpox disease. In 2007, the FDA approved the ACAM2000 immunization for individuals who are at high risk of smallpox. ACAM2000 is also believed to induce some cross-protective immunity against mpox [17,18]. The JYNNEOS vaccine was approved by the FDA in 2019, administered subcutaneously in a two-dose series, 0.5 mL per dose at 4 weeks apart. Later, in 2022, emergency-use authorization (EUA) was issued for intradermal administration in a two-dose series, 0.1 mL per dose, 4 weeks apart, to increase the supply of vaccine [19]. Concurrently, in August 2022, the head of Indonesia’s National Agency of Drug and Food Control authorized the emergency use of the JYNNEOS vaccine.

Data in this study were collected from August 2022 to December 2023. This study aims to describe the epidemiological data, clinical features, and mortality of mpox patients. In addition, this study aims to provide a comprehensive analysis by identifying and examining the differences both epidemiologically and clinically between mpox patients who are living with HIV (PLHIV) and those who are not (non-PLHIV). Through this approach, this study seeks to find any variations in the disease’s progression, symptoms, and transmission patterns between these two groups, thereby contributing to a deeper understanding of how mpox affects individuals with different immunological statuses.

## 2. Material and Methods

### 2.1. Study Design and Population

This study used a retrospective study design to determine the epidemiology, clinical features, and outcomes of MPXV infection and the differences between PLHIV and non-PLHIV. The inclusion criteria in this study were patients aged above 17 years old who resided in greater Jakarta between August 2022 and December 2023, who were confirmed as mpox by polymerase chain reaction (PCR) examination, and who were treated in Jakarta healthcare facilities, both as outpatients and inpatients. The exclusion criteria were patients who resided outside greater Jakarta and those who were not treated in Jakarta healthcare facilities. The samples were taken by whole sampling methods, where the entire population in the research is used as the sample.

### 2.2. Data Collection

Data were obtained from the epidemiological and clinical investigation form of mpox cases provided by the Indonesian Ministry of Health (Figure S1), which is integrated into the reporting system in the Special Capital Region of Jakarta Health District, and we also collected our sample HIV data from the Indonesian HIV/AIDS Information System. The data collection involved 25 primary healthcare facilities and 17 hospitals with reported mpox cases in Jakarta.

### 2.3. Variables Collected

We collected character demography data such as assigned sex at birth, age, occupation, HIV status, Anti-retroviral Therapy (ART), total CD4, and viral load; we also collected clinical manifestations; complications; outcome; treatment; isolation period; and laboratory examination data.

### 2.4. Data Analysis

These data were coded to be subsequently analyzed. All the categorical variables were analyzed for the frequencies and the percentages, while the continuous variables were analyzed for mean and SD.

## 3. Results

**Figure 1:**
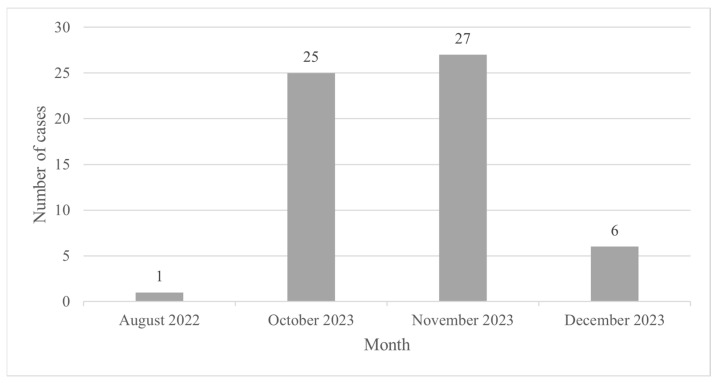
Number of reported mpox cases in Jakarta per month.

Out of 59 cases, 50 patients reportedly resided in Jakarta, while 9 patients resided in cities near to Jakarta (Figure S2). Most of the cases were reported from South Jakarta, with a total of 19 cases (Figure S2). According to the report by the Special Capital Region of Jakarta Health District, the first case was found in August 2022, then another new case emerged in October 2023, and the number of mpox cases peaked in November 2023 (Figure 1). The majority of the cases (74.6%) were reported by primary healthcare, while 25.4% of the cases were reported by hospitals.

In this study, all the cases were male (59 cases) and most of them were MSM, comprising 91.5% of the population. Some of the MSM population were also admitted as bisexual (9/54, 16.6%). The population’s mean age was 30.3 years old (21–49, SD 5.310). The majority of the cases (46/59, 78.0%) were PLHIV, with 39/46 patients (84.8%) already having received treatment with ART before their diagnosis of mpox. Two (3.4%) patients just received their HIV infection diagnosis after being diagnosed with mpox. There were three patients (6.5%) whose ART status were unknown. We were only able to obtain 18 results for total CD4 and 21 results for viral load. Most of the identified total CD4 from PLHIV with ART were 200–500 cells/mm^3^, while the PLHIV without ART total CD4 was mostly <200 cells/mm^3^. Thirteen non-PLHIV were found in the population (13/59, 22%).

About 40 (40/59, 67.8%) cases were reported to have had a sexual intercourse history in the last 21 days, and 29 (29/40, 72.5%) of them were confirmed to be PLHIV, while 11 (11/40, 27.5%) cases were non-PLHIV. None of the non-PLHIV patients had a history of consuming pre-exposure prophylaxis. Marital status was not recorded in this study. Thus, there were no data on spouse tracing. 

There were five confirmed cases reported with a history of traveling abroad. Two reportedly visited countries in Europe, two patients visited Malaysia, and one patient visited China. Further demographic data can be seen in Table 1.

Clinical manifestations reported in mpox cases may vary. Signs and symptoms were separated in this study. Out of 59 mpox cases, the most frequently reported symptoms were skin lesions (93.2%), followed by fever, defined as the patient’s body temperature > 37.5 °C (84.3%), and rash (66.1%). Lymphadenopathy was also a common finding in mpox patients and, in this study, mostly manifested in the inguinal area (39.0%). 

In general, most skin lesions were located on the face (64.4%), lower extremities (44.1%), and palm (42.4%). Out of seven (11.9%) patients who manifested perioral lesions, three out of seven (42.9%) patients reportedly had mucosal manifestations inside the mouth.

Most lesion morphologies were vesicles (52.5%) and pustules (49.2%) (Figure S3B). There was a difference in the lesion morphology tendencies in PLHIV who had received ART and those who had not received ART. In PLHIV, more than half of PLHIV with ART manifested with vesicle (56.0%) and pustule (59.0%) lesions, while PLHIV without ART all manifested with necrotic lesions (Figure S3A). Half of the PLHIV without ART showed vesicles, umbilicated pustules, and ulcers as their most common skin lesion manifestations. 

The majority of the patients (62.7%) had <25 lesions. There was also a difference in the number of lesions between PLHIV with ART and without ART: the majority of PLHIV with ART (25/39, 64.0%) had lesions <25, while PLHIV without ART (3/4, 75.0%) mostly had 25 to 99 lesions. Another important characteristic of a lesion was its pain and pruritus. Most cases reportedly had pain in the lesions, which comprised 54.2% of the cases. In contrast, only 5.1% of patients complained about pruritus in the lesion. Other clinical manifestations can be seen in Table 2.

Based on the clinical manifestations, only nine patients (15.3%) were classified as severe mpox according to the Indonesian guideline for the prevention and control of mpox, five patients were reported to have cervical lymphadenopathy and complained of dysphagia, two patients had more than 250 lesions, one patient complained of vomiting and nausea, and one patient was classified as severe, due to eye pain and laboratory findings. Most of these patients were PLHIV (7/9, 77.8%). There were two (3.4%) cases that have experienced organ involvement such as gastrointestinal involvement, vision involvement, and cellulitis around the perianal area due to mpox. 

Our study showed that 23/59 cases (39.0%) were concurrently diagnosed with sexually transmitted infection (STI), 22 patients were diagnosed with syphilis and 17/22 (77.3%) of them were PLHIV. Only one (1.7%) patient was diagnosed with HSV, and they were also PLHIV. Other than STIs, TB was also found among the patients. There were three patients (5.1%) with TB history and two patients presented with active TB (3.4%). Other comorbidities that were also reported were hypertension (3.4%), liver disease (3.4%), malignancy (1.7%), diabetes (1.7%), and other comorbidities (5.1%) such as pneumonia, colitis caused by Cytomegalovirus, and immune reconstitution inflammatory syndrome.

All of the cases were examined by PCR for laboratory confirmation of their specimens. None of the patients were tested using serology testing. Based on the result, PLHIV showed a higher percentage of positive results when using necrotic lesions as the specimen (100%). In comparison, non-PLHIV showed a higher percentage of positive results when using lesion fluid as the specimen (100%). (Table 3).

From the 51 data we successfully collected, the median interval for isolation is 20 days, with the shortest isolation period having been 6 days and the longest having been 46 days. Our patients were predominantly isolated in the hospital (55.9%), while the rest (44.1%) were self-isolated at home. 

Two (3.4%) severe mpox patients reportedly died due to HIV-related opportunistic infection, not mpox. Neither patient had initiated antiretroviral therapy (ART) and both patient’s CD4 counts were below 200 cells/mm^3^. Their viral load data were unavailable due to the Indonesian policy that viral load testing would only proceed after consuming ART for six months. One of the patients already received tecovirimat for 14 days, and was previously given cidofovir.

## 4. Discussion

Since the first case emerged in 2022, the government had taken measures regarding the disease by raising mpox awareness among healthcare and health workers on how to diagnose and manage mpox. Thus, the case finding and management of mpox became better, and we found 59 confirmed cases in Jakarta and analyzed them. Our analysis finds the majority of the mpox patient characteristics are male and in the productive age (mean age 30.3 years old, SD 5.310). A large portion of the cases are reported to have a history of sexual contact with men 21 days before the diagnosis. Mpox is mostly transmitted through direct skin-to-skin and mucosal contact during sexual activities. However, transmission via sperm should be studied further. This aligns with a previous study in 16 countries in 2022, where most patients diagnosed with mpox were identified among MSM (98.0%) of patients in a report of 528 cases from 16 countries, and many reported high-risk sexual behaviors as a potential risk factor [20].

Between PLHIV and non-PLHIV, both mostly manifested skin lesions (93.2%), fever (81.4%), rashes (66.1%), and lymphadenopathy (59.3%). A rash is a skin discoloration that has not yet developed into primary lesions. The confirmed mpox patients not showing skin lesions were those found from contact tracing and those who came by themselves for screening. We found similar findings to other studies in Nigeria and China, in that fever and rashes were the most common manifestation of mpox [21,22]. Most skin lesions manifested on the face (64.4%), lower extremities (44.1%), and palm (42.4%). The characteristics are somewhat similar to the study from Nigeria in 2023, where the most common regions were the face (93.0%), lower extremities (81.0%), trunk (79.0%), and palms (61.0%) [22].

Both PLHIV and non-PLHIV manifested vesicles (52.5%) and pustules (49.2%) were the most common skin lesion morphology. However, our study finds that there was a different trend in lesion morphology between PLHIV who had received ART and those who had not received ART. PLHIV with ART mostly manifested vesicle and pustule lesions, while PLHIV without ART lesions were mostly manifested with necrotic lesions (4/4, 100%); the percentage was higher if we compared to PLHIV with ART, with only 26% (10/39) of them manifesting necrotic lesions. This is an intriguing result, since the lesion progressions from rash to pustules took approximately a week for each stage to develop and it took 2–4 weeks to develop necrotic lesion shedding from the onset [23]. While a lot of the PLHIV without ART in our population presented with necrotic lesion, we do not have the data on how long the PLHIV without ART would take to develop necrotic lesion, and, therefore, the progression of the lesion in PLHIV without ART needs to be studied in the future.

A study stated that PLHIV with CD4 counts <200 cells/mm^3^ usually shows a severe necrotizing lesion [24]. These data suit our findings, since all CD4 count of PLHIV without ARV are under 200 cells/mm^3^, and most of them are showing necrotic lesions. Therefore, we should be giving more attention to patients who present with a more advanced lesion, because the patients could be HIV patients with low CD4 count and need more attention for the treatment.

Concurrent STIs were also common findings in MPXV infections; about 37.2% of our cases were diagnosed with syphilis. This number is higher than in other studies in the UK, where syphilis was found in 11% of the population [25]. All of our patients were also examined using PCR, and the highest positive rate was from using necrotic lesions (100%) and lesion fluid (94.0%) as the specimens. Considering the high positive rate in the lesion sample, the amount of HIV population in this study, and also the findings of syphilis in the population, it is very possible that the spread of mpox in Jakarta was most likely due to close contact, especially when they had engaged in sexual activity.

In the beginning, Indonesia regulated that all mpox patients should be isolated in hospitals, but the recent policy stated that self-isolation at home was allowed if the case did not require hospital aid. Therefore, our hospitalization rate is higher (55.9%) than in other studies, such as the study from Germany, which has a low hospitalization number (4.0%), and where the patients were hospitalized only if there was a severe clinical manifestation [26].

Indonesia places significant emphasis on mpox preventive measures, providing MVA-BN (JYNNEOS) vaccine in a limited supply targeted for high-risk populations, such as healthcare workers and MSM with a sexual contact history in the last two weeks. A registration and screening form for high-risk populations has been provided to facilitate the vaccination process (Figure S4). However, some high-risk patients did not comply, as there were only 86.9% who completed the second dose of the vaccine [4]. There might be some hesitancy to take the second dose of the vaccine. The cause of incompliance with the second dose in the Indonesian population has not been studied yet. According to the study by Agroia et al. (2023), it might be due to some patients being unaware of the risk of not completing the doses. Another reason might be the accessibility of the vaccines [27]. In Indonesia, the vaccines are only available at selected healthcare facilities and with selected schedules only. Meanwhile, the reach of mpox cases is extensive, thus creating challenges in access for patients residing outside Jakarta who are unable to return to Jakarta to receive the second dose of the vaccine. This implies that adequate education regarding mpox is required, especially regarding vaccination, together with an integrated vaccination system in the Greater Jakarta area to increase coverage and accessibility. These measures would not only be beneficial to those who are highly at risk, but would also protect the low-risk population, such as those who are not practicing risky sexual behavior and who are non-MSM.

Our study still has several limitations, such as the limited availability of our laboratory data, which can be attributed to the fact that not all patients underwent the same set of examinations. Laboratory examinations are not uniformly standardized across all receiving laboratories. Consequently, the number of data varied across different specimens and the results were not reported thoroughly. Additionally, since the epidemiological forms were completed by various healthcare professionals across different facilities, there is a possibility that some of the information provided may be subjective. As mpox is an emerging disease, we have conducted several mpox trainings for healthcare professionals; however, it this training has not reached all healthcare professionals effectively, leading to inconsistent reports due to differences in interpretation and reporting practices.

## 5. Conclusions

Mpox is an infectious re-emerging disease that affects Indonesia. In this study, the majority of the identified mpox cases were found among male PLHIV in productive age and the MSM community. Syphilis was found as a concomitant infection in this group. Notably, severe manifestations of mpox were predominantly seen in PLHIV who were not receiving ART, indicating that the absence of treatment could exacerbate the disease’s severity. Given these findings, it is crucial to establish a strong surveillance system and conduct thorough clinical examinations to ensure early detection, effective management, and prevention of mpox, particularly in high-risk populations such as PLHIV. Considering vaccines also play a part in the prevention of mpox, it is a requirement to educate the high-risk population and healthcare workers regarding vaccination. These approaches will be essential to reduce the impact of mpox and prevent further transmission.

## Figures and Tables

**Table 1:** Demography characteristics of the cases described in this study.

Characteristics	Number of Participants (*n* = 59, %)	HIV (*n* = 46)			Non-HIV (*n* = 13, %)
		Received ART (*n* = 39, %)	Without ART (*n* = 4, %)	ART Unknown * (*n* = 3, %)	
Assigned Sex at Birth					
Male	59 (100)	39 (100)	4 (100)	3 (100)	13 (100)
Female	0 (0)	0 (0)	0 (0)	0 (0)	0 (0)
Age in Years	Mean: 30.3 (21–49, SD 5.310)				
18–24	7 (11.9)	4 (10.3)	2 (50.0)	0 (0)	1 (7.7)
25–29	21 (35.6)	15 (38.0)	1 (25.0)	1 (33.3)	4 (30.8)
30–39	29 (49.2)	18 (46.0)	1 (25.0)	2 (66.7)	8 (61.5)
40–49	2 (3.4)	2 (5.0)	0 (0)	0 (0)	0 (0)
MSM/Non-MSM/Unknown					
MSM	54 (91.5)	37 (94.8)	4 (100)	3 (100)	10 (76.9)
Non-MSM	4 (6.8)	2 (5.0)	0 (0)	0 (0)	2 (15.4)
Unknown	1 (1.7)	0 (0)	0 (0)	0 (0)	1 (1.7)
Occupation					
Sex worker	3 (5.1)	3 (8.0)	0 (0)	0 (0)	0 (0)
Other	53 (89.8)	33 (85.0)	4 (100)	3 (100)	13 (100)
Unknown	3 (5.1)	3 (8.0)	0 (0)	0 (0)	0 (0)
Total CD4 (cells/mm^3^)					
<200	6 (13)	3 (8.0)	3 (75.0)	0 (0)	NA
200–500	8 (17.4)	8 (21.0)	0 (0)	0 (0)	NA
>500	4 (8.7)	4 (10.0)	0 (0)	0 (0)	NA
Unknown **	28 (60.9)	24 (61.5)	1 (25.0)	3 (100)	NA
Viral Load (copies/mL)					
≥40	1 (2)	1 (3.0)	0 (0)	0 (0)	NA
<40	20 (43.5)	20 (51.3)	0 (0)	0 (0)	NA
Unknown ***	25 (54.3)	18 (46.2)	4 (100)	3 (100)	NA

* At the time of Mpox diagnosis. ** Patient with no CD4 data available. *** Patient with no viral load data available. NA: Not Applicable.

**Table 2:** Clinical manifestation of mpox patients.

Clinical Manifestations	Number of Participants (*n* = 59, %)	HIV (*n* = 46)			Non-HIV (*n* = 13, %)
		Received ART (*n* = 39, %) *	Without ART (*n* = 4, %) *	ART Unknown (*n* = 3, %) *	
Skin Lesion	55 (93.2)	36 (92.0)	4 (100)	2 (66.7)	13 (100)
Site of Lesion					
Face	38 (64.4)	25 (64.0)	4 (100)	2 (66.7)	7 (53.8)
Lower extremities	26 (44.1)	15 (38.0)	3 (75.0)	1 (33.3)	7 (53.8)
Palm	25 (42.4)	15 (38.0)	3 (75.0)	0 (0)	7 (53.8)
Genital	21 (35.6)	14 (36.0)	1 (25.0)	0 (0)	6 (46.2)
Chest	20 (33.9)	14 (36.0)	3 (75.0)	1 (33.3)	2 (15.4)
Body	16 (27.1)	9 (23.0)	2 (50.0)	0 (0)	5 (38.5)
Perianal	12 (20.3)	8 (21.0)	2 (50.0)	0 (0)	2 (15.4)
Foot	9 (15.3)	6 (15.0)	0 (0)	0 (0)	3 (23.1)
Perioral	7 (11.9)	4 (10.0)	2 (50.0)	0 (0)	1 (7.7)
Other	19 (32.2)	11 (28.0)	2 (50.0)	0 (0)	6 (46.2)
Lesion morphology					
Vesicle	31 (52.5)	22 (56.0)	2 (50.0)	1 (33.3)	6 (46.2)
Pustule	29 (49.2)	23 (59.0)	0 (0)	1 (33.3)	5 (38.5)
Necrotic Lesion	18 (30.5)	10 (26.0)	4 (100)	1 (33.3)	3 (23.1)
Papule	18 (30.5)	12 (31.0)	1 (25.0)	1 (33.3)	4 (30.8)
Umbilicated Pustule	10 (16.9)	6 (15.0)	2 (50.0)	0 (0)	2 (15.4)
Ulcer	6 (10.2)	3 (8.0)	2 (50.0)	1 (33.3)	1 (7.7)
Macule	4 (6.8)	3 (8.0)	0 (0)	1 (33.3)	1 (7.7)
Other	2 (3.4)	1 (3.0)	1 (25.0)	0 (0)	0 (0)
Number of Lesions					
<25 (Benign)	37 (62.7)	25 (64.0)	0 (0)	2 (66.7)	10 (76.9)
25–99 (Medium)	11 (18.6)	7 (18.0)	2 (50.0)	0 (0)	2 (15.4)
>250 (Very Severe)	2 (3.4)	1 (3.0)	1 (25.0)	0 (0)	0 (0)
Unknown	9 (15.3)	6 (15.4)	1 (25.0)	1 (33.3)	1 (7.7)
Pain in lesion	32 (54.2)	21 (53.8)	2 (50.0)	1 (33.3)	8 (61.5)
Lesion pruritus	3 (5.1)	1 (3.0)	1 (25.0)	0 (0)	1 (7.7)
Fever	48 (81.4)	31 (79.5)	4 (100)	2 (66.7)	11 (84.6)
Rash	39 (66.1)	25 (64.1)	3 (75.0)	1 (33.3)	10 (76.9)
Lymphadenopathy	35 (59.3)	23 (59.0)	2 (50.0)	1 (33.3)	9 (69.2)
Inguinal	23 (39.0)	15 (38.0)	1 (25.0)	1 (33.3)	6 (46.2)
Cervical	15 (25.4)	9 (23.0)	1 (25.0)	0 (0)	5 (38.5)
Axilla	3 (5.1)	3 (8.0)	0 (0)	0 (0)	0 (0)
Other	1 (1.7)	1 (3.0)	0 (0)	0 (0)	0 (0)
Sore throat	25 (42.4)	15 (38.5)	1 (25.0)	2 (66.7)	7 (53.8)
Myalgia	23 (39.0)	14 (36.0)	2 (50.0)	1 (33.3)	6 (46.2)
Shiver	19 (32.2)	11 (28.0)	1 (25.0)	1 (33.3)	6 (46.2)
Anogenital pain	19 (32.2)	15 (38.0)	1 (25.0)	0 (0)	3 (23.1)
Fatigue	14 (23.7)	8 (21.0)	2 (50.0)	0 (0)	4 (30.8)
Arthralgia	14 (23.7)	10 (26.0)	1 (25.0)	1 (33.3)	2 (15.4)
Swallowing pain	15 (25.4)	9 (23.0)	1 (25.0)	1 (33.3)	4 (30.8)
Respiratory symptoms	13 (22.0)	7 (18.0)	1 (25.0)	1 (33.3)	4 (30.8)
Genital inflammation	15 (25.4)	11 (21.0)	1 (25.0)	0 (0)	3 (23.1)
Asthenia	10 (16.9)	6 (15.0)	0 (0)	1 (33.3)	3 (23.1)
Backpain	10 (16.9)	8 (21.0)	0 (0)	0 (0)	2 (15.4)
Diarrhea	9 (15.3)	4 (10.0)	0 (0)	0 (0)	5 (38.5)
Nausea	10 (16.9)	7 (18.0)	0 (0)	0 (0)	3 (23.1)
Anogenital hemorrhage	7 (11.9)	5 (13.0)	1 (25.0)	0 (0)	1 (1.7)
Pain in mouth	8 (13.6)	4 (10.0)	1 (25.0)	1 (33.3)	2 (15.4)
Dysphagia	8 (13.6)	4 (10.0)	0 (0)	1 (33.3)	3 (5.1)
Vomiting	2 (3.4)	2 (5.0)	0 (0)	0 (0)	0 (0)
Eye redness	3 (5.1)	1 (3.0)	1 (25.0)	1 (33.3)	0 (0)
Others	5 (8.5)	5 (13.0)	0 (0)	0 (0)	0 (0)

* At the time of Mpox diagnosis.

**Table 3:** Specimens for PCR examination.

Specimens	Number of Specimens (*n* = 59, %)	Positive Rate (%)		
		Total	HIV	Non-HIV
Tonsilopharyngeal swab	56 (94.9)	84 (47/56)	84 (36/43)	85 (11/13)
Lesion fluid	53 (89.8)	96 (51/53)	95 (38/40)	100 (13/13)
Rectal swab	34 (57.6)	47 (13/34)	50 (13/26)	38 (3/8)
Necrotic lesion swab	10 (16.9)	100 (10/10)	100 (9/9)	100 (1/1)
Anogenital swab	9 (15.3)	56 (5/9)	50 (3/6)	67 (2/3)

## Data Availability

The details of the data in this study cannot be accessed publicly, due to the medical records policy in Indonesia.

## Supplementary Materials

The following supporting information can be downloaded at: https://www.mdpi.com/article/10.3390/vaccines13030210/s1. Figure S1: Epidemiological and clinical investigation form of mpox cases. Figure S2: Distribution of mpox case in Greater Jakarta. Figure S3: clinical appearance of Mpox clinical symptoms [28]. Figure S4: Mpox vaccination screening form [29].
