# Supplementary material for: Epidemiology and Clinical Features of Mpox in Jakarta, Indonesia, August 2022–December 2023
Source: Vaccines (Basel). 2025 Feb 20;13(3):210. doi: 10.3390/vaccines13030210 (PMC11945424; doi:10.3390/vaccines13030210)
Supplement: Supplementary file 1 [file vaccines-13-00210-s001.zip › S1.pdf]

1 ## Lampiran 4. Formulir Penyelidikan Epidemiologi dan Klinis Kasus *Mpox*

2 ## I. INFORMASI KASUS

A. Informasi Petugas Wawancara

Nama Fasyankes	:	Tanggal Wawancara	:
Tempat Tugas	:	HP Pewawancara	:
Nama Pewawancara	:	Tanggal Pelaporan	:

3

B. Informasi Pasien

Nama Pasien	:		Kriteria Pasien:	€ Suspek<br>€ Probable<br>€ Konfirmasi
NIK Pasien	:			
Nama Orang Tua/ KK	:			
No. HP	:			
Tgl Lahir Pasien	:	Umur: 18 tahun, 1 bulan		
Jenis Kelamin Pasien	: x Laki-laki	☐ Perempuan	Informan dalam wawancara ini	**x** Pasien sendiri<br>☐ Keluarga pasien
Pekerjaan Pasien	: ☐ Tenaga Kesehatan	x Lainnya, sebutkan		
Alamat Domisili Pasien	Jalan/Blok	:		
Alamat Domisili Pasien	RT/RW	:	Kabupaten/Kota	: Jakarta Utara
	Desa/Kelurahan	:	Telepon/HP	: ...
Orientasi Seksual	☐ Heteroseksual<br>☐ LSL (Laki-laki seks dengan laki-laki)<br>☐ Lesbian		☐ Biseksual<br>☐ Lainnya<br>x Tidak Diketahui	
Pekerja Seks	☐ Ya x Tidak ☐ Tidak Diketahui			

4

C. Status Pasien Saat Ini

Status Pasien Saat Ini : ☐ Sembuh ☐ Dalam Perawatan…………… ☐ Meninggal, tanggal : ...

5

D. Informasi Klinis Pasien

BB : 34 (kg) TB: 156 (cm)

Tanggal pertama kali timbul gejala: ...

Gejala	Ya/Tidak/Tidak Tahu
Demam	: x Ya ☐ Tidak ☐ Tidak Tahu<br>Bila iya sebutkan onset: tanggal/bulan/tahun 26/10/2023. suhu 38.2^{o}C
Ruam	: ☐ Ya x Tidak ☐ Tidak Tahu<br>Bila iya sebutkan onset: tanggal/bulan/tahun…………./……./……..
Lesi	: x Ya ☐ Tidak ☐ Tidak Tahu<br>Jika Ya, jumlah total lesi: ☐ 1-5 x 6-25 ☐ 26-100 ☐101-250 ☐ >250<br>x wajah, jumlah >10 ☐ seluruh badan, jumlah ….<br>☐ kaki, jumlah …. ☐ dada, jumlah, ….<br>☐ telapak kaki, jumlah …. ☐ telapak tangan, jumlah ….<br>x genital,jumlah …. ☐ perianal, jumlah ….<br>☐ mulut, jumlah ……..<br>x lainnya …….

	Apakah lesi dalam kondisi berkembang pada badan<br>☐ Ya x Tidak ☐ Tidak Tahu<br>Apakah lesi tersebut dalam dan jelas?<br>☐ Ya ☐ Tidak ☐ Tidak Tahu<br>Apakah lesi tersebut mempunyai ukuran dan kondisi perkembangan sama<br>☐ Ya ☐ Tidak x Tidak Tahu<br>Tipe Lesi, sebutkan:<br>☐ Makula x Umbilikasi pustula<br>☐ Papula ☐ Ulkus<br>☐ Vesikel x Krusta/Keropeng<br>☐ Pustula x Lainnya: vesikel<br>Nyeri pada lesi<br>x Ya ☐ Tidak ☐ Tidak Tahu
Pembengkakan kelenjar getah bening (limfadenopati)	x Ya ☐ Tidak ☐ Tidak Tahu<br>Jika Ya,<br>Inguinal: : ☐ Ya x Tidak ☐ Tidak Tahu<br>Axilla : ☐ Ya x Tidak ☐ Tidak Tahu<br>Cervical : ☐ Ya x Tidak ☐ Tidak Tahu<br>Lokasi Lainnya:....

Sakit tenggorokan	: ☐ Ya x Tidak ☐ Tidak Tahu	Nyeri di anogenital	: ☐ Ya x Tidak ☐ Tidak Tahu
Batuk/gejala respirasi	: ☐ Ya x Tidak ☐ Tidak Tahu	Perdarahan anogenital	: ☐ Ya x Tidak ☐ Tidak Tahu
Menggigil	: x Ya ☐ Tidak ☐ Tidak Tahu	Radang di genital	: ☐ Ya x Tidak ☐ Tidak Tahu
Asthenia	: ☐ Ya x Tidak ☐ Tidak Tahu	Nyeri di mulut	: x Ya ☐ Tidak ☐ Tidak Tahu
Athralgia	: ☐ Ya x Tidak ☐ Tidak Tahu	Sakit menelan	: ☐ Ya x Tidak ☐ Tidak Tahu
Myalgia	: ☐ Ya x Tidak ☐ Tidak Tahu	Susah menelan	: ☐ Ya x Tidak ☐ Tidak Tahu
Gejala pada mata (kemerahan, nyeri, dll)	: ☐ Ya x Tidak ☐ Tidak Tahu	Mual	: ☐ Ya x Tidak ☐ Tidak Tahu
Fatigue	: x Ya ☐ Tidak ☐ Tidak Tahu	Muntah	: ☐ Ya x Tidak ☐ Tidak Tahu
Backpain	: ☐ Ya x Tidak ☐ Tidak Tahu	Gejala lain sebutkan............	
Diare	: ☐ Ya x Tidak ☐ Tidak Tahu		

6

E. Kondisi Penyerta (Komorbid)

Hamil	: ☐ Ya x Tidak<br>Jika Ya, Minggu Gestasi: ... minggu<br>☐ Pos-partum (<6 minggu)	PPOK	: ☐ Ya ☐ Tidak
Diabetes	: ☐ Ya x Tidak	Penyakit Ginjal	: ☐ Ya x Tidak
Penyakit jantung	: ☐ Ya x Tidak	Penyakit Hati	: ☐ Ya x Tidak
Hipertensi	: ☐ Ya x Tidak	Tuberkulosis aktif	: ☐ Ya x Tidak
Keganasan	: ☐ Ya x Tidak	Tuberkulosis lampau	: ☐ Ya x Tidak
HIV	: ☐ Ya ☐ Tidak Belum tahu<br>Jika Ya:<br>☐ ARV ☐ Non-ARV	Bersamaan IMS lain	: ☐ Ya x Tidak<br>Jika Ya:<br>☐ Gonorrhea ☐ Syphilis

CD4 bila ada : ………

☐ HSV ☐ Klamidia
☐ Limfomagranolum
☐ Lainnya, Lain-lain sebutkan : …….

Gangguan Kekebalan Tubuh : x Ya, karena penyakit
☐ Ya, karena pengobatan
☐ Ya, alasan tidak diketahui
☐ Tidak
☐ Tidak diketahui

7
8

F. Komplikasi Klinis

Apakah kasus mengalami komplikasi
x Tidak ada
☐ ARDS (*Acute respiratory distress syndrome*)
☐ Infeksi Saluran Pernapasan Bawah (misal: pneumonia)
☐ Ensefalitis
☐ Meningoensefalitis
☐ Miokarditis
☐ Infeksi Kornea
☐ Sepsis
☐ Abses retrofaringeal
☐ Still birth (IUD)
☐ Infeksi sekunder
☐ Tidak diketahui
☐ Lainnya, sebutkan …..

9

G. Derajat Keparahan dan Hasil Akhir Pengobatan

Apakah kasus dirawat di rumah sakit? : x Ya ☐ Tidak

*Bila Ya
Nama RS terakhir :
Tanggal masuk RS terakhir :
Ruang rawat :
Perawatan ICU ☐ Ya x Tidak
Tindakan perawatan : …

Jika ada, nama-nama RS sebelumnya :

Status Pasien Terakhir ☐ Selesai isolasi / Sembuh x Masih dirawat ☐ Lost to Follow Up
☐ Meninggal, tanggal : ____ / ____ / __________

Kriteria Akhir Pasien
☐ Konfirmasi
x Suspek
☐ Probable
☐ Discarded

10

H. Riwayat Vaksinasi

Apakah memiliki Riwayat vaksin cacar (*smallpox*) : ☐ Ya x Tidak ☐ Tidak Tahu

Apakah ada hasil serologi orthopoxvirus : ☐ Ya x Tidak ☐ Tidak Tahu
Bila Ya hasil…………

Apakah memiliki Riwayat vaksin *mpox*? : ☐ Ya x Tidak ☐ Tidak Tahu
Jika Ya, jenis vaksin:
☐ Primary preventive (pre-exposure) vaccination (PPV)
☐ Post-exposure preventive vaccination (PEPV)

Dosis ke Merk Tanggal Pemberian

□ 1
□ 2

II. INVESTIGASI PAPARAN

I. Riwayat Paparan

Dalam 21 hari sebelum sakit, apakah memiliki riwayat perjalanan dari negara/wilayah terjangkit yang sedang terjadi KLB/melaporkan kasus *mpox*/ endemis? : □ Ya x Tidak □ Tidak Tahu

Negara	Kota	Maskapai	No. Kursi	No. Paspor	Tanggal Perjalanan	Tanggal Tiba di Tujuan (Kota/Indonesia)

Dalam 21 hari sebelum sakit, apakah memiliki kontak dengan kasus probable/konfirmasi? : □ Ya x Tidak □ Tidak Tahu

Nama	Umur	Jenis Kelamin	Alamat Rumah	No HP/telp yang dapat dihubungi	Tanggal kontak terakhir	Jenis kontak

Hubungan dengan kasus

□ Pasangan
□ Anggota Serumah
□ Kerabat
□ Teman
□ Pasangan Seksual
□ Fasilitas Kesehatan
□ Lainnya, Sebutkan……………………..

Jenis Kontak

□ kontak erat (<1m tanpa ada kontak fisik)
□ kontak fisik kulit ke kulit, tanpa kontak mukosa dan berhubungan seksual
□ Kontak mulut ke kulit (berciuman atau seks oral)
□ Kontak Berhubungan Seksual
□ Kontak dengan benda terkontaminasi (cairan, barang, sprei, dll) tanpa kontak langsung ke kasus
□ Tenaga kesehatan yang kontak dengan kasus tanpa APD yang sesuai
□ Tidak diketahui
□ Lainnya, sebutkan …….

Frekuensi kontak : □ Satu kali □ Beberapa kali
Total Durasi Kontak : □ <5 menit □ 5-15 menit □ 15 menit-1 jam □ 1-4 jam □ >4 jam

Detail Paparan

□ Rumah Tangga
Bila di Rumah Tangga, berapa orang yang tinggal dalam 1 rumah? Sebutkan …. orang
□ Hotel atau penginapan
□ Tempat kerja
□ Sekolah/tempat penitipan anak
□ Fasilitas Layanan Kesehatan (termasuk laboratorium)
□ Klub malam/pesta *private*/sauna dengan kontak seksual
□ Bar/restoran atau event kecil tanpa kontak seksual
□ Event besar tanpa kontak seksual (misal festival atau acara olahraga) *indoor*
□ Event besar tanpa kontak seksual (misal festival atau acara olahraga) *outdoor*

☐ Event besar dengan kontak seksual
☐ Tidak tahu
☐ Lainnya

Nama dan alamat lokasi paparan: …….

Riwayat Aktivitas Seksual

Dalam 21 hari sebelum bergejala, apakah melakukan hubungan seksual?
• Ya • Tidak x Tidak Tahu
Jika Ya, pilih jenis pasangan seksual: • Perempuan • Laki-laki

Jumlah pasangan seksual dalam 21 hari terakhir
☐ …… orang ☐ Tidak bersedia menjawab ☐ Tidak diketahui

Memiliki pasangan anonim dalam 21 hari terakhir
☐ Ya ☐ Tidak

Jika kasus merupakan Tenaga Kesehatan yang terpapar di Fasilitas Layanan Kesehatan

Dalam 21 hari apakah kontak langsung dengan kasus probable/konfirmasi? ☐ Ya ☐ Tidak ☐ Tidak diketahui

Apakah selama kontak dengan kasus probable/konfirmasi, menggunakan APD yang sesuai?
☐ Ya
☐ Tidak
☐ Hanya memakai beberapa APD
☐ Tidak diketahui

APD yang digunakan
☐ Masker bedah
☐ Gaun
☐ Sarung tangan
☐ Respirator (misal N95, FFP2, dll)
☐ Pelindung mata

Apakah terjadi kerusakan APD saat digunakan? ☐ Ya ☐ Tidak ☐ Tidak diketahui

Apakah menerapkan 5 momen mencuci tangan? ☐ Ya ☐ Tidak ☐ Tidak diketahui

Riwayat Kontak Hewan

Dalam 21 hari terakhir apakah terdapat kontak dengan hewan (cairan/lesi/konsumsi daging)?
• Ya • Tidak x Tidak Tahu

Jika ya,
• Hewan peliharaan
• Hewan peliharaan pengerat (misal hamster, guinea pig, mencit, dll)
• Hewan liar (misal monyet, tupai pohon, dll)

Apakah memiliki hewan peliharaan? • Ya • Tidak • Tidak Tahu
Jika Ya, sebutkan jenis…..

Kemungkinan Mode Transmisi

☐ Kontak langsung dari orang ke orang (kecuali transmisi dari ibu ke anak selama kehamilan atau persalinan, penularan di layanan Kesehatan, atau transmisi seksual)
☐ Transmisi seksual
☐ Hewan ke manusia
☐ Penularan di layanan Kesehatan
☐ Penularan di laboratorium

☐ Penularan dari ibu ke anak selama kehamilan atau persalinan
☐ Benda terkontaminasi
☐ Penerima transfusi
☐ Tidak diketahui
x Lainnya, sebutkan:

Apakah ada orang lain yang mengalami sakit yang sama di rumah, tetangga, tempat kerja atau keluarga yang lain?	: ☐ Ya **x** Tidak ☐ Tidak Tahu<br>Jika Ya, lengkapi keterangan orang yang dimaksud

Nama	Umur	Jenis Kelamin	Hubungan dengan Kasus	Alamat Rumah	No HP/telp yang dapat dihubungi	Tanggal kontak terakhir	Jenis kontak

CATATAN (jika ada data, informasi apa saja yang dianggap perlu silakan ditulis

Perjalanan Penyakit
(waktu paparan, timbul gejala, pemeriksaan pendukung, rujukan dan sebagainya) digambarkan dalam garis waktu berikut:

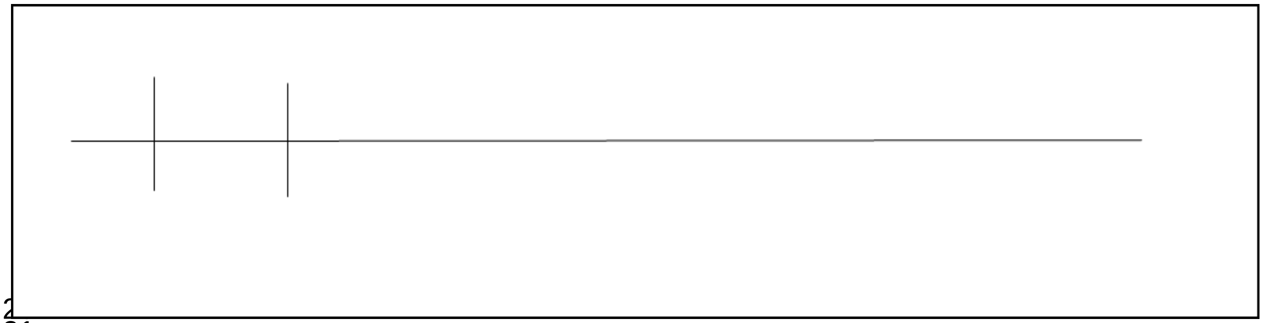

J. Informasi Pemeriksaan Penunjang

No.	**Jenis Spesimen**	**Tanggal pengambilan Sampel**	**Tempat Pemeriksaan**	**Hasil**
1	Caran lesi			
2	Keropeng/krusta			
3	Serum			
4	Swab anogenital			
5	Swab tonsil/orofaring			
6	Swab rektal			

7 Lain-lain, Sebutkan

22

23 **III. PEMANTAUAN KLINIS DAN INFORMASI LABORATORIUM**

J. Penilaian Lesi dan Tanda Vital

*DIISI JIKA MENJADI KASUS KONFIRMASI PADA SAAT FOLLOW UP HARIAN ATAU TIAP 3-5 HARI

Tanggal Penilaian	__/__/_____
Tanda Vital	Suhu: __ °C<br>Tekanan Darah: ____/____<br>Detak Nadi: ___/ ___<br>Laju pernapasan: ___/___
Lesi	: ☐ Ya ☐ Tidak ☐ Tidak Tahu<br>Jika Ya, jumlah total lesi: ☐ 1-5 ☐ 6-25 ☐ 26-100 ☐101-250 ☐ >250<br>☐ wajah, jumlah .... ☐ seluruh badan, jumlah ....<br>☐ kaki, jumlah .... ☐ dada, jumlah, ...<br>☐ telapak kaki, jumlah .... ☐ telapak tangan, jumlah ....<br>☐ genital, jumlah .... ☐ perianal, jumlah ....<br>☐ mulut, jumlah .... ☐ lainnya, jumlah ....<br>Tipe Lesi, sebutkan:<br>☐ Makula ☐ Umbilikasi pustula<br>☐ Papula ☐ Ulkus<br>☐ Vesikel ☐ Krusta/Keropeng<br>☐ Pustula ☐ Lainnya<br>Nyeri pada lesi<br>☐ Ya ☐ Tidak ☐ Tidak Tahu

24 * Tabel ini diperbanyak sesuai kebutuhan pemantauan (harian/ tiap 3-5 hari)

25

K. Komplikasi Klinis*

*DIISI JIKA MENJADI KASUS KONFIRMASI PADA SAAT VISIT TERAKHIR, MENINGGAL, DIRUJUK, SEMBUH

Apakah kasus mengalami komplikasi	☐ Tidak ada<br>☐ ARDS (Acute respiratory distress syndrome)<br>☐ Infeksi Saluran Pernapasan Bawah (misal: pneumonia)<br>☐ Ensefalitis<br>☐ Meningoensefalitis<br>☐ Miokarditis<br>☐ Infeksi Kornea	☐ Sepsis<br>☐ Abses retrofaringeal<br>☐ Still birth (IUD)<br>☐ Infeksi sekunder<br>☐ Tidak diketahui<br>☐ Lainnya, sebutkan .....

26

L. Pemeriksaan Laboratorium*

*DIISI JIKA MENJADI KASUS KONFIRMASI PADA SAAT PERTAMA PERAWATAN, VISIT TERAKHIR, MENINGGAL, DIRUJUK, SEMBUH

Tanggal Pemeriksaan	________	________	Tanggal Pemeriksaan	________	________
Laboratorium	**Nilai**	**Nilai**	**Laboratorium**	**Nilai**	**Nilai**
ALT (U/L)			Glukosa (mg/dL)		

AST (U/L)	Laktat (mmol/L)
Kreatinin (µmol/L)	Hemoglobin (g/L)
Potasium (mEg/L)	Bilirubin total (mg/dL)
Urea (mmol/L)	WBC Count (cells x 10^9/L)
Kreatinin Kinase (U/L)	Platelet (x 10^9/L)
Kalsium (mg/dL)	PT
Natrium (mEq/L)	aPTT
CRP (mg/dL)	Lainnya
Serologi mpox	
Hasil Pemeriksaan *Genomic Sequencing*	

27

M. Pengobatan*

*DIISI JIKA MENJADI KASUS KONFIRMASI PADA SAAT VISIT TERAKHIR, MENINGGAL, DIRUJUK, SEMBUH

Cairan Oral/Orogastric ☐ Ya ☐ Tidak ☐ Tidak Diketahui

Cairan Intravena ☐ Ya ☐ Tidak ☐ Tidak Diketahui

Eksperimental orthopox antiviral ☐ Ya ☐ Tidak ☐ Tidak Diketahui

Antiviral	Tanggal Pertama Diberikan	Dosis	Frekuensi	Rute IV/PO/IM	Durasi
☐ Tecovirimat					
☐ Brincidovir					
☐ Cidofovir					
☐ Lainnya					

Antibakteri ☐ Ya ☐ Tidak ☐ Tidak Diketahui

Antibakteri	Tanggal Pertama Diberikan	Dosis	Frekuensi	Rute IV/PO/IM	Durasi
☐ Amoxicillin-clavulanic					
☐ Ceftriaxone					
☐ Lainnya					

Antifungal ☐ Ya ☐ Tidak ☐ Tidak Diketahui

Antifungal	Tanggal Pertama Diberikan	Dosis	Frekuensi	Rute IV/PO/IM	Durasi
☐ Fluconazole					
☐ Lainnya					

28

L. Perawatan Suportif*

*DIISI JIKA MENJADI KASUS KONFIRMASI SELAMA MENJALANI PERAWATAN DI RUMAH SAKIT

ICU	☐ Ya ☐ Tidak ☐ Tidak Diketahui<br>Jika Ya, durasi hari ……<br>Tanggal masuk ICU :<br>Tanggal keluar ICU :
Terapi Oksigen	☐ Ya ☐ Tidak ☐ Tidak Diketahui<br>Jika Ya, durasi hari ……<br>Oxygen flow:<br>☐1–5 L/min ☐6–10 L/min ☐11–15 L/min ☐ > 15 L/min<br><br>Interface:<br>☐Nasal prongs ☐HF nasal cannula ☐Mask ☐Mask with reservoir ☐CPAP/NIV
Non-invasive Ventilation (misalnya BiPAP, CPAP)	☐ Ya ☐ Tidak ☐ Tidak Diketahui<br>Jika Ya, durasi hari ……
Invasive Ventilation	☐ Ya ☐ Tidak ☐ Tidak Diketahui<br>Jika Ya, durasi hari ……
Extracorporeal (ECMO) support	☐ Ya ☐ Tidak ☐ Tidak Diketahui<br>Jika Ya, durasi hari ……
Inotropes/vasopressors	☐ Ya ☐ Tidak ☐ Tidak Diketahui<br>Jika Ya, durasi hari ……
Renal replacement therapy (RRT) or dialysis?	☐ Ya ☐ Tidak ☐ Tidak Diketahui

IV. PELACAKAN KONTAK

M. Daftar Kontak Erat*

Diidentifikasi sejak kasus mulai gejala sampai dengan keropeng mengelupas/hilang (masa infeksius)

No	Nama	Umur	Jenis Kelamin	Hubungan dengan Kasus	Alamat Rumah	No HP/telp yang dapat dihubungi	Tanggal kontak terakhir	Tempat Kontak**	Jenis kontak***
1									
2									
3									
4									
5									

Ket: *diisi jika kriteria suspek, probable dan konfirmasi

**seperti rumah tangga, tempat kerja, sekolah, layanan Kesehatan, bar, restoran, sauna, klab malam, dan sebagainya

***Seperti pasangan seksual, anggota rumah tangga, teman kerja, dan lain-lain
