# Supplementary material for: Epidemiology and Clinical Features of Mpox in Jakarta, Indonesia, August 2022–December 2023
Source: Vaccines (Basel). 2025 Feb 20;13(3):210. doi: 10.3390/vaccines13030210 (PMC11945424; doi:10.3390/vaccines13030210)
Supplement: Supplementary file 1 [file vaccines-13-00210-s001.zip › S4_REV.pdf]

Figure S4: Mpox vaccination screening form.

Formulir pendaftaran dan penapisan vaksinasi monkeypox

* Required

1

Apakah anda ingin/setuju mendapatkan vaksinasi monkeypox? *

Anda akan dinilai apakah memenuhi syarat untuk mendapatkan vaksinasi Monkeypox. Diharapkan untuk mengisi formulir dengan sebenar-benarnya.

Jika memenuhi syarat, anda akan divaksinasi monkeypox sebanyak 2 kali dengan jeda 1 bulan.

- ◯ Setuju
- ◯ Tidak Setuju

Never give out your password. Report abuse

Microsoft 365

This content is created by the owner of the form. The data you submit will be sent to the form owner. Microsoft is not responsible for the privacy or security practices of its customers, including those of this form owner. Never give out your password.

Microsoft Forms| AI-Powered surveys, quizzes and polls Create my own form

Privacy and cookies | Consumer Health Privacy | Terms of use

Formulir pendaftaran dan penapisan vaksinasi monkeypox

* Required

Formulir penapisan (Data Diri)

2

Nama Sesuai KTP *

Enter your answer

3

Nomor Induk Kependudukan (NIK) *

Enter your answer

4

No Handphone (Aktif WA) *

Enter your answer

5

email

Enter your answer

6

Tempat tinggal/domisili *

- ◯ Jakarta Pusat
- ◯ Jakarta Utara
- ◯ Jakarta Barat
- ◯ Jakarta Selatan
- ◯ Jakarta Timur
- ◯ Bogor
- ◯ Depok
- ◯ Tangerang

◯ Bekasi

Never give out your password. Report abuse

Microsoft 365

This content is created by the owner of the form. The data you submit will be sent to the form owner. Microsoft is not responsible for the privacy or security practices of its customers, including those of this form owner. Never give out your password.

Microsoft Forms| AI-Powered surveys, quizzes and polls Create my own form

Privacy and cookies | Consumer Health Privacy | Terms of use

Formulir pendaftaran dan penapisan vaksinasi monkeypox

* Required

Formulir penapisan (Faktor Risiko)

7

Jenis Kelamin *

- (selected) Laki-Laki
- Perempuan
- Transpuan

8

Apakah jenis kelamin padangan seksual Anda? *

- (selected) Laki-Laki
- Perempuan
- Keduanya

9

Apakah anda aktif berhubungan seksual dalam 2 minggu terakhir? *

- (selected) Ya
- Tidak

10

Apakah dalam 2 minggu terakhir anda berhubungan seksual dengan selain pasangan tetap anda? *

- (selected) Iya, Pernah
- Tidak Pernah

11

Menurut anda, Apakah pasangan seksual anda memiliki pasangan seksual lain selain anda? *

- (selected) Iya
- Tidak

Tidak Tahu

12

Dalam 2 minggu terakhir, Berapa banyak pasangan seksual anda? *

isi dengan angka

13

Apakah anda pernah berhubungan seksual dengan orang yang memiliki gejala Monkey Pox? *

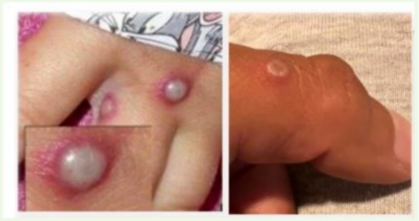

Jumlah lenting seperti di gambar biasanya ditemukan dalam jumlah sedikit (umumnya 1 - 25 lenting) di beberapa tempat di bagian tubuh, terasa nyeri. Gejala lain antara lain demam, sakit kepala, nyeri tulang dan otot, pembesaran kelenjar getah bening, nyeri tenggorok.

- (x) Pernah
- ( ) Tidak Penah

14

Apakah anda menggunakan kondom saat berhubungan seksual? *

- (x) Selalu
- ( ) Kadang-kadnag
- ( ) Tidak Pernah

15

Apakah anda mengetahui status HIV anda? *

- (x) ODHIV
- ( ) Bukan ODHIV
- ( ) Tidak tahu / Belum pernah periksa

16

Nama Layanan PDP akses ARV *

Contoh : Puskesmas Tanah Abang atau RS Carolus

Enter your answer

17

Hasil Viral Load Terkahir *

- ○ Tidak Terdeteksi
- ◉ Terdeteksi

18

Apakah anda memiliki pendamping dari komunitas *

- ◉ Ya, didampingi
- ○ Tidak ada

19

Sebutkan siapa komunitas yang mendapingi anda?

sebutkan nama pendamping atau instansi

Enter your answer

Never give out your password. Report abuse

Microsoft 365

This content is created by the owner of the form. The data you submit will be sent to the form owner. Microsoft is not responsible for the privacy or security practices of its customers, including those of this form owner. Never give out your password.

Microsoft Forms| AI-Powered surveys, quizzes and polls Create my own form

Privacy and cookies | Consumer Health Privacy | Terms of use

Formulir pendaftaran dan penapisan vaksinasi monkeypox

Pemilihan Jadwal Vaksinasi

Jika anda terpilih menjadi peserta Vaksin Monkeypox, pilihlah jadwal dan lokasi vaksin

17

Lokasi vaksin

- ◯ Jakarta Pusat
- ◯ Jakarta Barat
- ◯ Jakarta Selatan
- ◯ Jakarta Timur

18

Jadwal Vaksin

- ◯ Pagi (10.00 - 12.00)
- ◯ Siang (13.00 - 15.00)
- ◯ Sore (16.00 - 18.00)

Never give out your password. Report abuse

Microsoft 365

This content is created by the owner of the form. The data you submit will be sent to the form owner. Microsoft is not responsible for the privacy or security practices of its customers, including those of this form owner. Never give out your password.

Microsoft Forms| AI-Powered surveys, quizzes and polls Create my own form

Privacy and cookies | Consumer Health Privacy | Terms of use
