# Supplementary material for: Epidemiology and Clinical Features of Mpox in Jakarta, Indonesia, August 2022–December 2023
Source: Vaccines (Basel). 2025 Feb 20;13(3):210. doi: 10.3390/vaccines13030210 (PMC11945424; doi:10.3390/vaccines13030210)
Supplement: Supplementary file 1 [file vaccines-13-00210-s001.zip › Supplementary materials.pdf]

Figure S1: Epidemiological and clinical investigation form of mpox cases

Lampiran 4. Formulir Penyelidikan Epidemiologi dan Klinis Kasus *Mpox*
I. INFORMASI KASUS

A. Informasi Petugas Wawancara

Nama Fasyankes	:	Tanggal Wawancara	:
Tempat Tugas	:	HP Pewawancara	:
Nama Pewawancara	:	Tanggal Pelaporan	:

B. Informasi Pasien

Nama Pasien	:		Kriteria Pasien:	€ Suspek
NIK Pasien	:			€ Probable
Nama Orang Tua/ KK	:			€ Konfirmasi
No. HP	:			
Tgl Lahir Pasien	:	Umur: 18 tahun, 1 bulan		
Jenis Kelamin Pasien	: x Laki-laki	□ Perempuan	Informan dalam wawancara ini	**x** Pasien sendiri
Pekerjaan Pasien	: □ Tenaga Kesehatan	x Lainnya, sebutkan		□ Keluarga pasien
Alamat Domisili Pasien	Jalan/Blok	:		
Alamat Domisili Pasien	RT/RW	:	Kabupaten/Kota	: Jakarta Utara
	Desa/Kelurahan	:	Telepon/HP	: ...
Orientasi Seksual	□ Heteroseksual		□ Biseksual	
	□ LSL (Laki-laki seks dengan laki-laki)		□ Lainnya	
	□ Lesbian		x Tidak Diketahui	
Pekerja Seks	□ Ya x Tidak □ Tidak Diketahui			

C. Status Pasien Saat Ini

Status Pasien Saat Ini : □ Sembuh □ Dalam Perawatan.............. □ Meninggal, tanggal : ...

D. Informasi Klinis Pasien

BB : 34 (kg) TB: 156 (cm)

Tanggal pertama kali timbul gejala: ...

Gejala	Ya/Tidak/Tidak Tahu
Demam	: x Ya □ Tidak □ Tidak Tahu<br>Bila iya sebutkan onset: tanggal/bulan/tahun 26/10/2023. suhu 38.2^{o}C
Ruam	: □ Ya x Tidak □ Tidak Tahu<br>Bila iya sebutkan onset: tanggal/bulan/tahun............./......./........
Lesi	: x Ya □ Tidak □ Tidak Tahu<br>Jika Ya, jumlah total lesi: □ 1-5 x 6-25 □ 26-100 □101-250 □ >250<br>x wajah, jumlah >10 □ seluruh badan, jumlah ....<br>□ kaki, jumlah .... □ dada, jumlah, ...<br>□ telapak kaki, jumlah .... □ telapak tangan, jumlah ....<br>x genital,jumlah .... □ perianal, jumlah ....<br>□ mulut, jumlah ....<br>x lainnya ....<br>Apakah lesi dalam kondisi berkembang pada badan<br>□ Ya x Tidak □ Tidak Tahu<br>Apakah lesi tersebut dalam dan jelas?<br>□ Ya □ Tidak □ Tidak Tahu<br>Apakah lesi tersebut mempunyai ukuran dan kondisi perkembangan sama<br>□ Ya □ Tidak x Tidak Tahu<br>Tipe Lesi, sebutkan:<br>□ Makula x Umbilikasi pustula

	☐ Papula ☐ Ulkus<br>☐ Vesikel x Krusta/Keropeng<br>☐ Pustula x Lainnya: vesikel<br>Nyeri pada lesi<br>x Ya ☐ Tidak ☐ Tidak Tahu
Pembengkakan kelenjar getah bening (limfadenopati)	x Ya ☐ Tidak ☐ Tidak Tahu<br>Jika Ya,<br>Inguinal: : ☐ Ya x Tidak ☐ Tidak Tahu<br>Axilla : ☐ Ya x Tidak ☐ Tidak Tahu<br>Cervical : ☐ Ya x Tidak ☐ Tidak Tahu<br>Lokasi Lainnya:....

Sakit tenggorokan	: ☐ Ya x Tidak ☐ Tidak Tahu	Nyeri di anogenital	: ☐ Ya x Tidak ☐ Tidak Tahu
Batuk/gejala respirasi	: ☐ Ya x Tidak ☐ Tidak Tahu	Perdarahan anogenital	: ☐ Ya x Tidak ☐ Tidak Tahu
Menggigil	: x Ya ☐ Tidak ☐ Tidak Tahu	Radang di genital	: ☐ Ya x Tidak ☐ Tidak Tahu
Asthenia	: ☐ Ya x Tidak ☐ Tidak Tahu	Nyeri di mulut	: x Ya ☐ Tidak ☐ Tidak Tahu
Athralgia	: ☐ Ya x Tidak ☐ Tidak Tahu	Sakit menelan	: ☐ Ya x Tidak ☐ Tidak Tahu
Myalgia	: ☐ Ya x Tidak ☐ Tidak Tahu	Susah menelan	: ☐ Ya x Tidak ☐ Tidak Tahu
Gejala pada mata (kemerahan, nyeri, dll)	: ☐ Ya x Tidak ☐ Tidak Tahu	Mual	: ☐ Ya x Tidak ☐ Tidak Tahu
Fatigue	: x Ya ☐ Tidak ☐ Tidak Tahu	Muntah	: ☐ Ya x Tidak ☐ Tidak Tahu
Backpain	: ☐ Ya x Tidak ☐ Tidak Tahu	Gejala lain sebutkan............	
Diare	: ☐ Ya x Tidak ☐ Tidak Tahu		

E. Kondisi Penyerta (Komorbid)

Hamil	: ☐ Ya x Tidak<br>Jika Ya, Minggu Gestasi: ... minggu<br>☐ Pos-partum (<6 minggu)	PPOK	: ☐ Ya ☐ Tidak
Diabetes	: ☐ Ya x Tidak	Penyakit Ginjal	: ☐ Ya x Tidak
Penyakit jantung	: ☐ Ya x Tidak	Penyakit Hati	: ☐ Ya x Tidak
Hipertensi	: ☐ Ya x Tidak	Tuberkulosis aktif	: ☐ Ya x Tidak
Keganasan	: ☐ Ya x Tidak	Tuberkulosis lampau	: ☐ Ya x Tidak
HIV	: ☐ Ya ☐ Tidak Belum tahu<br>Jika Ya:<br>☐ ARV ☐ Non-ARV<br>CD4 bila ada :	Bersamaan IMS lain	: ☐ Ya x Tidak<br>Jika Ya:<br>☐ Gonorrhea ☐ Syphilis<br>☐ HSV ☐ Klamidia<br>☐ Limfomagranolum<br>☐ Lainnya,
Gangguan Kekebalan Tubuh	: x Ya, karena penyakit<br>☐ Ya, karena pengobatan<br>☐ Ya, alasan tidak diketahui<br>☐ Tidak<br>☐ Tidak diketahui	Lain-lain sebutkan	: ....

F. Komplikasi Klinis

Apakah kasus mengalami komplikasi	x Tidak ada<br>☐ ARDS (*Acute respiratory distress syndrome*)<br>☐ Infeksi Saluran Pernapasan Bawah (misal: pneumonia)<br>☐ Ensefalitis<br>☐ Meningoensefalitis<br>☐ Miokarditis	☐ Sepsis<br>☐ Abses retrofaringeal<br>☐ Still birth (IUD)<br>☐ Infeksi sekunder<br>☐ Tidak diketahui<br>☐ Lainnya, sebutkan .....

□ Infeksi Kornea

G. Derajat Keparahan dan Hasil Akhir Pengobatan

Apakah kasus dirawat di rumah sakit? : x Ya □ Tidak

*Bila Ya
Nama RS terakhir :
Tanggal masuk RS terakhir :
Ruang rawat :
Perawatan ICU □ Ya x Tidak
Tindakan perawatan : ...

Jika ada, nama-nama RS sebelumnya :

Status Pasien Terakhir
□ Selesai isolasi / Sembuh x Masih dirawat □ Lost to Follow Up
□ Meninggal, tanggal : ____ / ____ / __________

Kriteria Akhir Pasien
□ Konfirmasi
x Suspek
□ Probable
□ Discarded

H. Riwayat Vaksinasi

Apakah memiliki Riwayat vaksin cacar (*smallpox*) : □ Ya x Tidak □ Tidak Tahu

Apakah ada hasil serologi orthopoxvirus : □ Ya x Tidak □ Tidak Tahu
Bila Ya hasil...........

Apakah memiliki Riwayat vaksin *mpox*? : □ Ya x Tidak □ Tidak Tahu
Jika Ya, jenis vaksin:
□ Primary preventive (pre-exposure) vaccination (PPV)
□ Post-exposure preventive vaccination (PEPV)

Dosis ke	Merk	Tanggal Pemberian
□ 1		
□ 2		

II. INVESTIGASI PAPARAN

I. Riwayat Paparan

Dalam 21 hari sebelum sakit, apakah memiliki riwayat perjalanan dari negara/wilayah terjangkit yang sedang terjadi KLB/melaporkan kasus *mpox*/ endemis? : □ Ya x Tidak □ Tidak Tahu

Negara	Kota	Maskapai	No. Kursi	No. Paspor	Tanggal Perjalanan	Tanggal Tiba di Tujuan (Kota/Indonesia)

Dalam 21 hari sebelum sakit, apakah memiliki kontak dengan kasus probable/konfirmasi? : □ Ya x Tidak □ Tidak Tahu

Nama	Umur	Jenis Kelamin	Alamat Rumah	No HP/telp yang dapat dihubungi	Tanggal kontak terakhir	Jenis kontak

Hubungan dengan kasus
□ Pasangan
□ Anggota Serumah
□ Kerabat
□ Teman
□ Pasangan Seksual
□ Fasilitas Kesehatan

Jenis Kontak
□ kontak erat (<1m tanpa ada kontak fisik)
□ kontak fisik kulit ke kulit, tanpa kontak mukosa dan berhubungan seksual
□ Kontak mulut ke kulit (berciuman atau seks oral)
□ Kontak Berhubungan Seksual

☐ Lainnya, Sebutkan……………………..	☐ Kontak dengan benda terkontaminasi (cairan, barang, sprei, dll) tanpa kontak langsung ke kasus<br>☐ Tenaga kesehatan yang kontak dengan kasus tanpa APD yang sesuai<br>☐ Tidak diketahui<br>☐ Lainnya, sebutkan …….<br><br>Frekuensi kontak : ☐ Satu kali ☐ Beberapa kali<br>Total Durasi Kontak : ☐ <5 menit ☐ 5-15 menit ☐ 15 menit-1 jam ☐ 1-4 jam ☐ >4 jam
Detail Paparan	☐ Rumah Tangga<br>Bila di Rumah Tangga, berapa orang yang tinggal dalam 1 rumah? Sebutkan …. orang<br>☐ Hotel atau penginapan<br>☐ Tempat kerja<br>☐ Sekolah/tempat penitipan anak<br>☐ Fasilitas Layanan Kesehatan (termasuk laboratorium)<br>☐ Klub malam/pesta *private*/sauna dengan kontak seksual<br>☐ Bar/restoran atau event kecil tanpa kontak seksual<br>☐ Event besar tanpa kontak seksual (misal festival atau acara olahraga) *indoor*<br>☐ Event besar tanpa kontak seksual (misal festival atau acara olahraga) *outdoor*<br>☐ Event besar dengan kontak seksual<br>☐ Tidak tahu<br>☐ Lainnya<br><br>Nama dan alamat lokasi paparan: …….
Riwayat Aktivitas Seksual	Dalam 21 hari sebelum bergejala, apakah melakukan hubungan seksual?<br>• Ya • Tidak x Tidak Tahu<br>Jika Ya, pilih jenis pasangan seksual: • Perempuan • Laki-laki<br><br>Jumlah pasangan seksual dalam 21 hari terakhir<br>☐ …… orang ☐ Tidak bersedia menjawab ☐ Tidak diketahui<br><br>Memiliki pasangan anonim dalam 21 hari terakhir<br>☐ Ya ☐ Tidak

Jika kasus merupakan Tenaga Kesehatan yang terpapar di Fasilitas Layanan Kesehatan	Dalam 21 hari apakah kontak langsung dengan kasus probable/konfirmasi?	☐ Ya ☐ Tidak ☐ Tidak diketahui
	Apakah selama kontak dengan kasus probable/konfirmasi, menggunakan APD yang sesuai?	☐ Ya<br>☐ Tidak<br>☐ Hanya memakai beberapa APD<br>☐ Tidak diketahui
	APD yang digunakan	☐ Masker bedah<br>☐ Gaun<br>☐ Sarung tangan<br>☐ Respirator (misal N95, FFP2, dll)<br>☐ Pelindung mata
	Apakah terjadi kerusakan APD saat digunakan?	☐ Ya ☐ Tidak ☐ Tidak diketahui
	Apakah menerapkan 5 momen mencuci tangan?	☐ Ya ☐ Tidak ☐ Tidak diketahui

Riwayat Kontak Hewan	Dalam 21 hari terakhir apakah terdapat kontak dengan hewan (cairan/lesi/konsumsi daging)?<br>• Ya • Tidak x Tidak Tahu<br><br>Jika ya,<br>• Hewan peliharaan<br>• Hewan peliharaan pengerat (misal hamster, guinea pig, mencit, dll)

• Hewan liar (misal monyet, tupai pohon, dll)

Apakah memiliki hewan peliharaan? • Ya • Tidak • Tidak Tahu
Jika Ya, sebutkan jenis.....

Kemungkinan Mode Transmisi	☐ Kontak langsung dari orang ke orang (kecuali transmisi dari ibu ke anak selama kehamilan atau persalinan, penularan di layanan Kesehatan, atau transmisi seksual)<br>☐ Transmisi seksual<br>☐ Hewan ke manusia<br>☐ Penularan di layanan Kesehatan<br>☐ Penularan di laboratorium<br>☐ Penularan dari ibu ke anak selama kehamilan atau persalinan<br>☐ Benda terkontaminasi<br>☐ Penerima transfusi<br>☐ Tidak diketahui<br>x Lainnya, sebutkan:
Apakah ada orang lain yang mengalami sakit yang sama di rumah, tetangga, tempat kerja atau keluarga yang lain?	: ☐ Ya **x** Tidak ☐ Tidak Tahu<br>Jika Ya, lengkapi keterangan orang yang dimaksud

Nama	Umur	Jenis Kelamin	Hubungan dengan Kasus	Alamat Rumah	No HP/telp yang dapat dihubungi	Tanggal kontak terakhir	Jenis kontak

CATATAN (jika ada data, informasi apa saja yang dianggap perlu silakan ditulis

Perjalanan Penyakit
(waktu paparan, timbul gejala, pemeriksaan pendukung, rujukan dan sebagainya) digambarkan dalam garis waktu berikut:

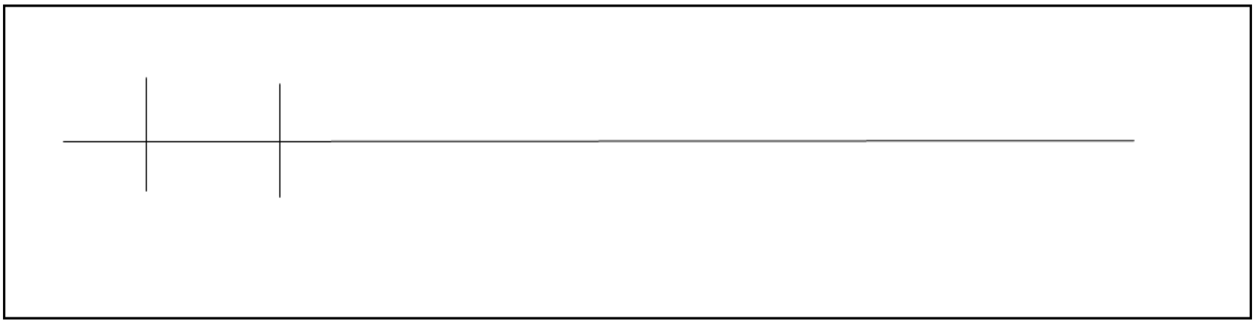

J. Informasi Pemeriksaan Penunjang

No.	**Jenis Spesimen**	**Tanggal pengambilan Sampel**	**Tempat Pemeriksaan**	**Hasil**
1	Caran lesi			
2	Keropeng/krusta			
3	Serum			
4	Swab anogenital			
5	Swab tonsil/orofaring			
6	Swab rektal			

7 Lain-lain, Sebutkan

III. PEMANTAUAN KLINIS DAN INFORMASI LABORATORIUM

J. Penilaian Lesi dan Tanda Vital

*DIISI JIKA MENJADI KASUS KONFIRMASI PADA SAAT FOLLOW UP HARIAN ATAU TIAP 3-5 HARI

Tanggal Penilaian	__/__/_____
Tanda Vital	Suhu: __ °C<br>Tekanan Darah: ____/____<br>Detak Nadi: ___/ ___<br>Laju pernapasan: ___/___
Lesi	: □ Ya □ Tidak □ Tidak Tahu<br>Jika Ya, jumlah total lesi: □ 1-5 □ 6-25 □ 26-100 □101-250 □ >250<br>□ wajah, jumlah .... □ seluruh badan, jumlah ....<br>□ kaki, jumlah .... □ dada, jumlah, ...<br>□ telapak kaki, jumlah .... □ telapak tangan, jumlah ....<br>□ genital, jumlah .... □ perianal, jumlah ....<br>□ mulut, jumlah .... □ lainnya, jumlah ....<br>Tipe Lesi, sebutkan:<br>□ Makula □ Umbilikasi pustula<br>□ Papula □ Ulkus<br>□ Vesikel □ Krusta/Keropeng<br>□ Pustula □ Lainnya<br>Nyeri pada lesi<br>□ Ya □ Tidak □ Tidak Tahu

* Tabel ini diperbanyak sesuai kebutuhan pemantauan (harian/ tiap 3-5 hari)

K. Komplikasi Klinis*

*DIISI JIKA MENJADI KASUS KONFIRMASI PADA SAAT VISIT TERAKHIR, MENINGGAL, DIRUJUK, SEMBUH

Apakah kasus mengalami komplikasi	□ Tidak ada<br>□ ARDS (Acute respiratory distress syndrome)<br>□ Infeksi Saluran Pernapasan Bawah (misal: pneumonia)<br>□ Ensefalitis<br>□ Meningoensefalitis<br>□ Miokarditis<br>□ Infeksi Kornea	□ Sepsis<br>□ Abses retrofaringeal<br>□ Still birth (IUD)<br>□ Infeksi sekunder<br>□ Tidak diketahui<br>□ Lainnya, sebutkan .....

L. Pemeriksaan Laboratorium*

*DIISI JIKA MENJADI KASUS KONFIRMASI PADA SAAT PERTAMA PERAWATAN, VISIT TERAKHIR, MENINGGAL, DIRUJUK, SEMBUH

Tanggal Pemeriksaan	________	________	Tanggal Pemeriksaan	________	________
Laboratorium	**Nilai**	**Nilai**	**Laboratorium**	**Nilai**	**Nilai**
ALT (U/L)			Glukosa (mg/dL)		
AST (U/L)			Laktat (mmol/L)		
Kreatinin (μmol/L)			Hemoglobin (g/L)		
Potasium (mEg/L)			Bilirubin total (mg/dL)		
Urea (mmol/L)			WBC Count (cells x 10^9/L)		
Kreatinin Kinase (U/L)			Platelet (x 10^9/L)		
Kalsium (mg/dL)			PT		
Natrium (mEq/L)			aPTT		

CRP (mg/dL) Lainnya

Serologi mpox

Hasil Pemeriksaan *Genomic Sequencing*

M. Pengobatan*

*DIISI JIKA MENJADI KASUS KONFIRMASI PADA SAAT VISIT TERAKHIR, MENINGGAL, DIRUJUK, SEMBUH

Cairan Oral/Orogastric ☐ Ya ☐ Tidak ☐ Tidak Diketahui

Cairan Intravena ☐ Ya ☐ Tidak ☐ Tidak Diketahui

Eksperimental orthopox antiviral ☐ Ya ☐ Tidak ☐ Tidak Diketahui

Antiviral	Tanggal Pertama Diberikan	Dosis	Frekuensi	Rute IV/PO/IM	Durasi
☐ Tecovirimat					
☐ Brincidovir					
☐ Cidofovir					
☐ Lainnya					

Antibakteri ☐ Ya ☐ Tidak ☐ Tidak Diketahui

Antibakteri	Tanggal Pertama Diberikan	Dosis	Frekuensi	Rute IV/PO/IM	Durasi
☐ Amoxicillin-clavulanic					
☐ Ceftriaxone					
☐ Lainnya					

Antifungal ☐ Ya ☐ Tidak ☐ Tidak Diketahui

Antifungal	Tanggal Pertama Diberikan	Dosis	Frekuensi	Rute IV/PO/IM	Durasi
☐ Fluconazole					
☐ Lainnya					

L. Perawatan Suportif*

*DIISI JIKA MENJADI KASUS KONFIRMASI SELAMA MENJALANI PERAWATAN DI RUMAH SAKIT

ICU ☐ Ya ☐ Tidak ☐ Tidak Diketahui
Jika Ya, durasi hari ……
Tanggal masuk ICU :
Tanggal keluar ICU :

Terapi Oksigen ☐ Ya ☐ Tidak ☐ Tidak Diketahui
Jika Ya, durasi hari ……
Oxygen flow:
☐1–5 L/min ☐6–10 L/min ☐11–15 L/min ☐ > 15 L/min

Interface:
☐Nasal prongs ☐HF nasal cannula ☐Mask ☐Mask with reservoir ☐CPAP/NIV

Non-invasive Ventilation (misalnya BiPAP, CPAP) ☐ Ya ☐ Tidak ☐ Tidak Diketahui
Jika Ya, durasi hari ……

Invasive Ventilation ☐ Ya ☐ Tidak ☐ Tidak Diketahui
Jika Ya, durasi hari ……

Extracorporeal (ECMO) support ☐ Ya ☐ Tidak ☐ Tidak Diketahui
Jika Ya, durasi hari ……

Inotropes/vasopressors ☐ Ya ☐ Tidak ☐ Tidak Diketahui

Jika Ya, durasi hari ......

Renal replacement therapy (RRT) or dialysis?	☐ Ya ☐ Tidak ☐ Tidak Diketahui

IV. PELACAKAN KONTAK

M. Daftar Kontak Erat*

Diidentifikasi sejak kasus mulai gejala sampai dengan keropeng mengelupas/hilang (masa infeksius)

No	Nama	Umur	Jenis Kelamin	Hubungan dengan Kasus	Alamat Rumah	No HP/telp yang dapat dihubungi	Tanggal kontak terakhir	Tempat Kontak**	Jenis kontak***
1									
2									
3									
4									
5									

Ket: *diisi jika kriteria suspek, probable dan konfirmasi

**seperti rumah tangga, tempat kerja, sekolah, layanan Kesehatan, bar, restoran, sauna, klab malam, dan sebagainya

***Seperti pasangan seksual, anggota rumah tangga, teman kerja, dan lain-lain

Figure S2: Distribution of mpox case in Greater Jakarta from August 2022 to December 2023

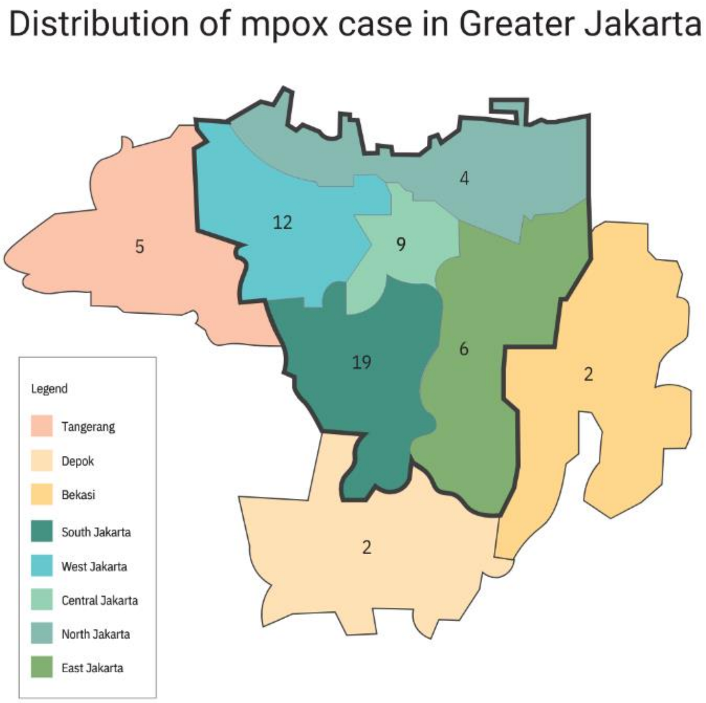

Figure S3A-B: clinical appearance of Mpox clinical symptoms reported by Sinto R, et al. (2024) [1]

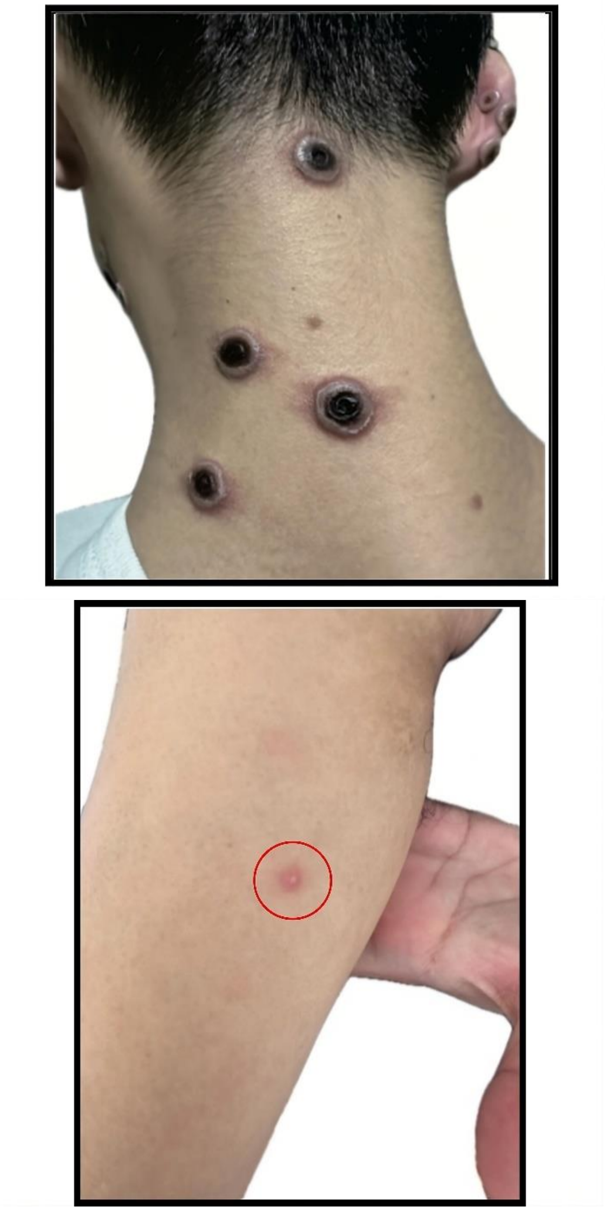

Figure S4: Mpox vaccination screening form. [2]

Formulir pendaftaran dan penapisan vaksinasi monkeypox

* Required

1

Apakah anda ingin/setuju mendapatkan vaksinasi monkeypox? *

Anda akan dinilai apakah memenuhi syarat untuk mendapatkan vaksinasi Monkeypox. Diharapkan untuk mengisi formulir dengan sebenar-benarnya.

Jika memenuhi syarat, anda akan divaksinasi monkeypox sebanyak 2 kali dengan jeda 1 bulan.

○ Setuju

○ Tidak Setuju

Never give out your password. Report abuse

Microsoft 365

This content is created by the owner of the form. The data you submit will be sent to the form owner. Microsoft is not responsible for the privacy or security practices of its customers, including those of this form owner. Never give out your password.

Microsoft Forms| AI-Powered surveys, quizzes and polls Create my own form

Privacy and cookies | Consumer Health Privacy | Terms of use

Formulir pendaftaran dan penapisan vaksinasi monkeypox

* Required

Formulir penapisan (Data Diri)

2

Nama Sesuai KTP *

Enter your

3

Nomor Induk Kependudukan (NIK) *

Enter your

4

No Handphone (Aktif WA) *

Enter your

5

email

Enter your

6

Tempat tinggal/domisili *

- Jakarta Pusat
- Jakarta Utara
- Jakarta Barat
- Jakarta Selatan
- Jakarta Timur
- Bogor
- Depok
- Tangerang

◯ Bekasi

Never give out your password. Report abuse

Microsoft 365

This content is created by the owner of the form. The data you submit will be sent to the form owner. Microsoft is not responsible for the privacy or security practices of its customers, including those of this form owner. Never give out your password.

Microsoft Forms| AI-Powered surveys, quizzes and polls Create my own form

Privacy and cookies | Consumer Health Privacy | Terms of use

Formulir pendaftaran dan penapisan vaksinasi monkeypox

* Required

Formulir penapisan (Faktor Risiko)

7

Jenis Kelamin *

- (●) Laki-Laki
- ( ) Perempuan
- ( ) Transpuan

8

Apakah jenis kelamin padangan seksual Anda? *

- (●) Laki-Laki
- ( ) Perempuan
- ( ) Keduanya

9

Apakah anda aktif berhubungan seksual dalam 2 minggu terakhir? *

- (●) Ya
- ( ) Tidak

10

Apakah dalam 2 minggu terakhir anda berhubungan seksual dengan selain pasangan tetap anda? *

- (●) Iya, Pernah
- ( ) Tidak Pernah

11

Menurut anda, Apakah pasangan seksual anda memiliki pasangan seksual lain selain anda? *

- (●) Iya
- ( ) Tidak

◯ Tidak Tahu

12

Dalam 2 minggu terakhir, Berapa banyak pasangan seksual anda? *

isi dengan angka

13

Apakah anda pernah berhubungan seksual dengan orang yang memiliki gejala Monkey Pox? *

Jumlah lenting seperti di gambar biasanya ditemukan dalam jumlah sedikit (umumnya 1 - 25 lenting) di beberapa tempat di bagian tubuh, terasa nyeri. Gejala lain antara lain demam, sakit kepala, nyeri tulang dan otot, pembesaran kelenjar getah bening, nyeri tenggorok.

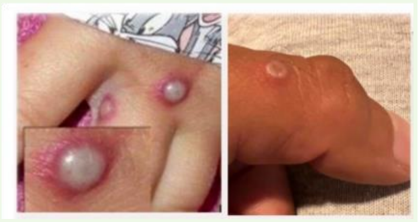

◉ Pernah

◯ Tidak Penah

14

Apakah anda menggunakan kondom saat berhubungan seksual? *

◉ Selalu

◯ Kadang-kadnag

◯ Tidak Pernah

15

Apakah anda mengetahui status HIV anda? *

◉ ODHIV

◯ Bukan ODHIV

◯ Tidak tahu / Belum pernah periksa

16

Nama Layanan PDP akses ARV *

Contoh : Puskesmas Tanah Abang atau RS Carolus

Enter your

17

Hasil Viral Load Terkahir *

○ Tidak Terdeteksi

◉ Terdeteksi

18

Apakah anda memiliki pendamping dari komunitas *

◉ Ya, didampingi

○ Tidak ada

19

Sebutkan siapa komunitas yang mendapingi anda?

sebutkan nama pendamping atau instansi

Enter your

Never give out your password. Report abuse

Microsoft 365

This content is created by the owner of the form. The data you submit will be sent to the form owner. Microsoft is not responsible for the privacy or security practices of its customers, including those of this form owner. Never give out your password.

Microsoft Forms| AI-Powered surveys, quizzes and polls Create my own form

Privacy and cookies | Consumer Health Privacy | Terms of use

Formulir pendaftaran dan penapisan vaksinasi monkeypox

Pemilihan Jadwal Vaksinasi

Jika anda terpilih menjadi peserta Vaksin Monkeypox, pilihlah jadwal dan lokasi vaksin

17

Lokasi vaksin

- ◯ Jakarta Pusat
- ◯ Jakarta Barat
- ◯ Jakarta Selatan
- ◯ Jakarta Timur

18

Jadwal Vaksin

- ◯ Pagi (10.00 - 12.00)
- ◯ Siang (13.00 - 15.00)
- ◯ Sore (16.00 - 18.00)

Never give out your password. Report abuse

Microsoft 365

This content is created by the owner of the form. The data you submit will be sent to the form owner. Microsoft is not responsible for the privacy or security practices of its customers, including those of this form owner. Never give out your password.

Microsoft Forms| AI-Powered surveys, quizzes and polls Create my own form

Privacy and cookies | Consumer Health Privacy | Terms of use

References:

1. Sinto, Robert, Johan, Alvin, Nilasari, Hanny, Yunihastuti, Evy, Nelwan, Erni J. Mpox skin lesion. Acta Med Indones-Indones J Intern Med. 2024;56.

2. Dinas Kesehatan Provinsi DKI Jakarta. Formulir pendaftaran dan penapisan vaksinasi monkeypox [Internet]. Available from: https://forms.office.com/r/XXY7shpyUS
