# Supplementary figures and images for: Epidemiology and Clinical Features of Mpox in Jakarta, Indonesia, August 2022–December 2023
Source: Vaccines (Basel). 2025 Feb 20;13(3):210. doi: 10.3390/vaccines13030210 (PMC11945424; doi:10.3390/vaccines13030210)

# Distribution of mpox case in Greater Jakarta

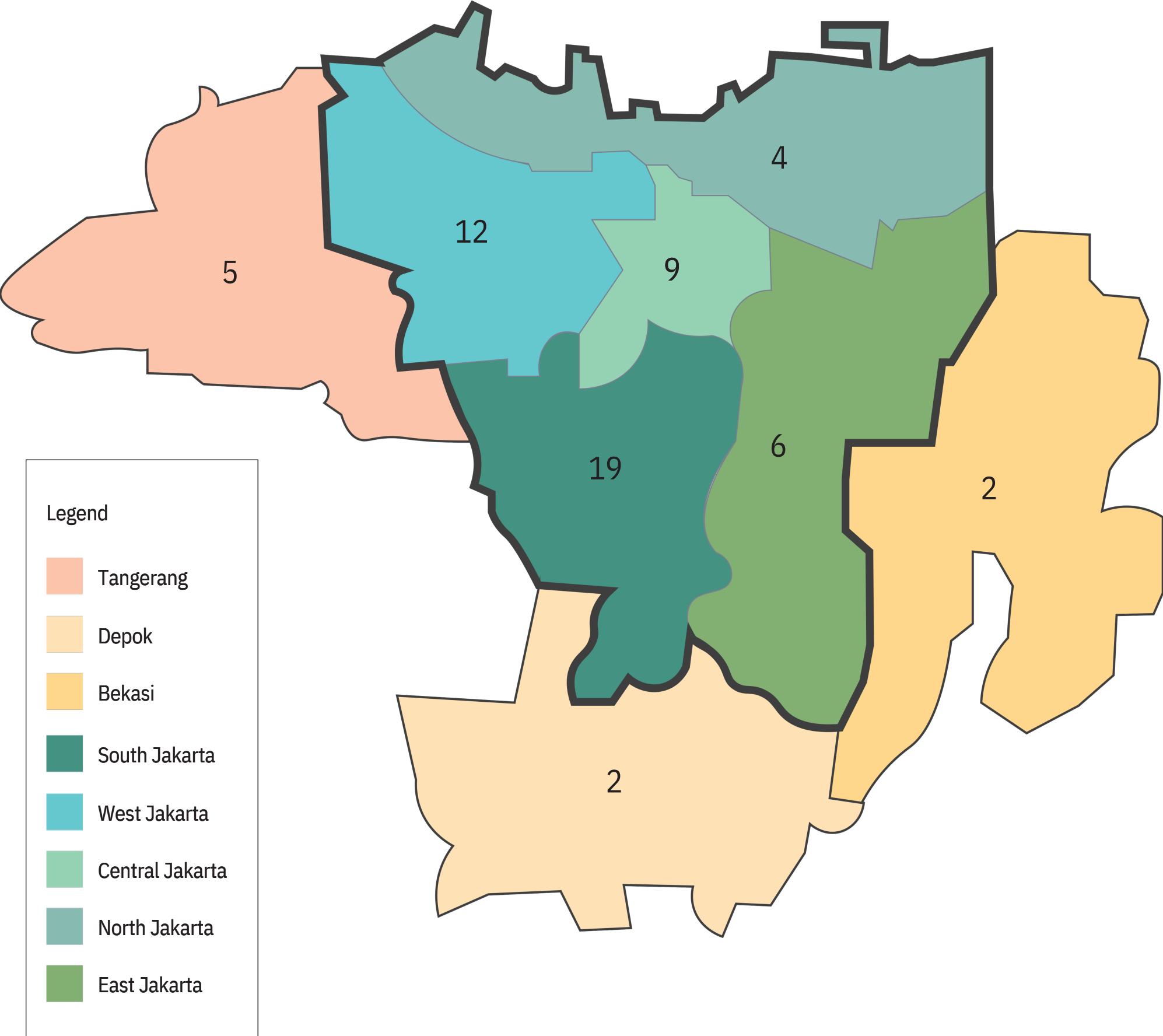

Supplement: Supplementary file 1 [file vaccines-13-00210-s001.zip › S2_Rev.pdf]

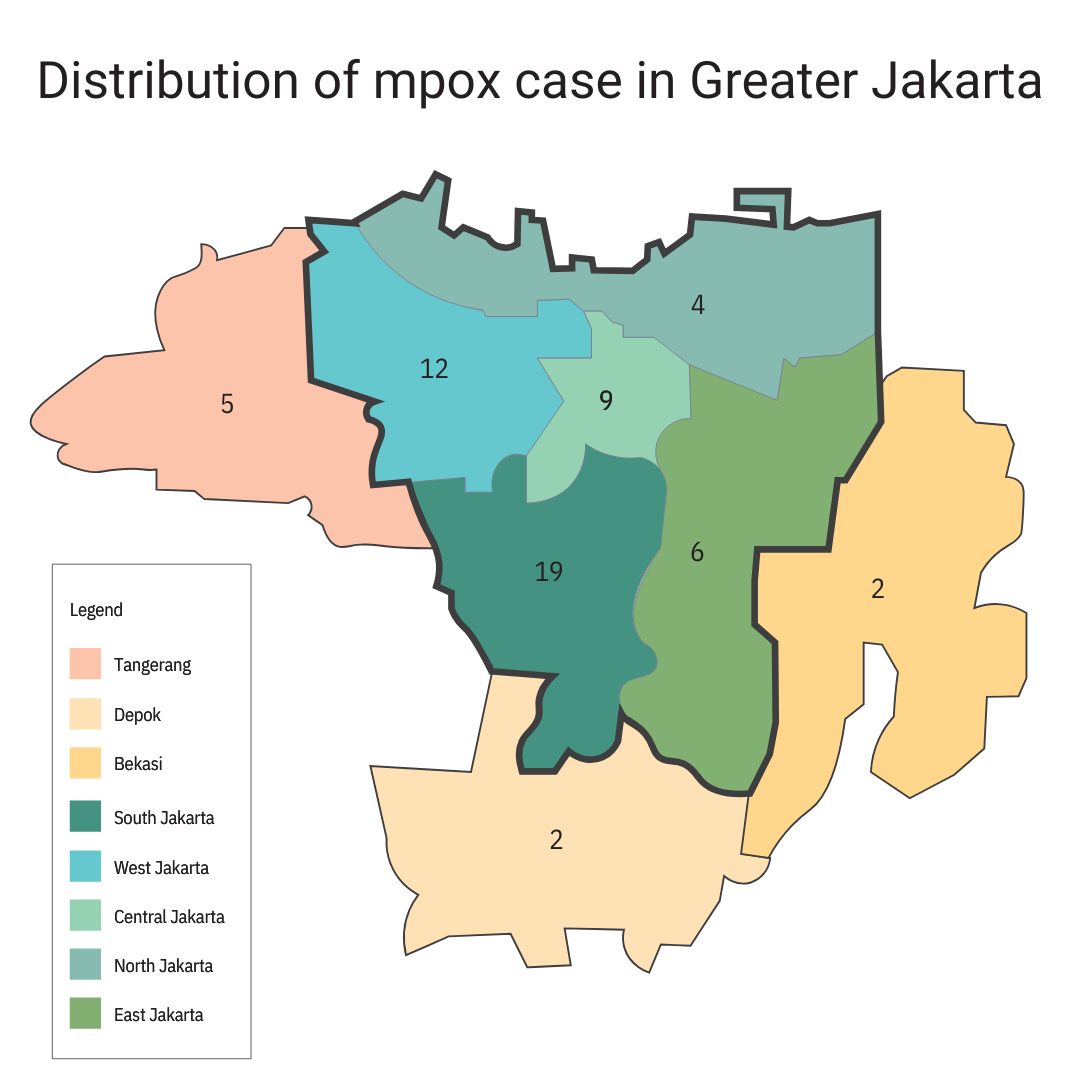

Supplement: Supplementary file 1 [file vaccines-13-00210-s001.zip › S2_Rev.png]

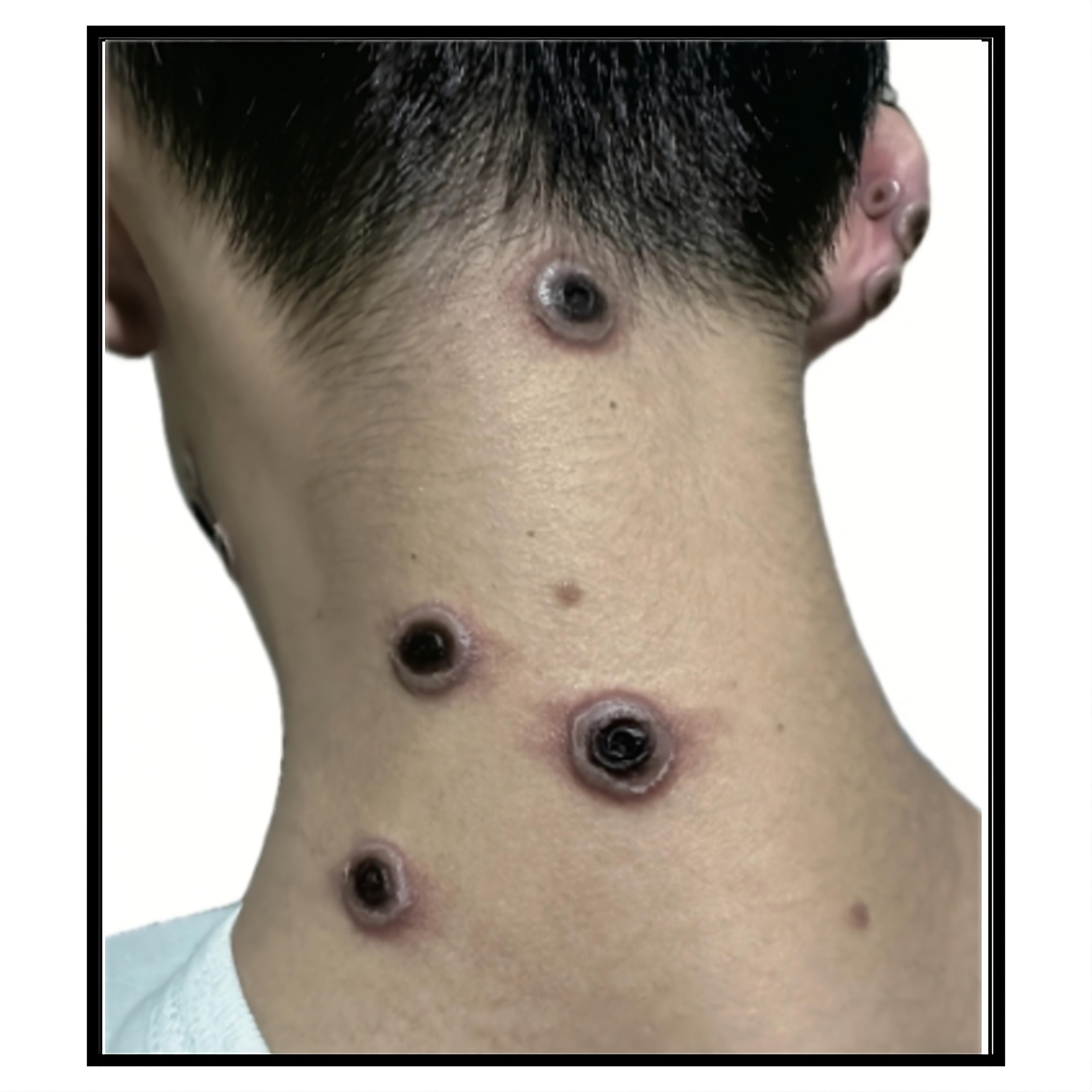

Supplement: Supplementary file 1 [file vaccines-13-00210-s001.zip › S3A.jpg]

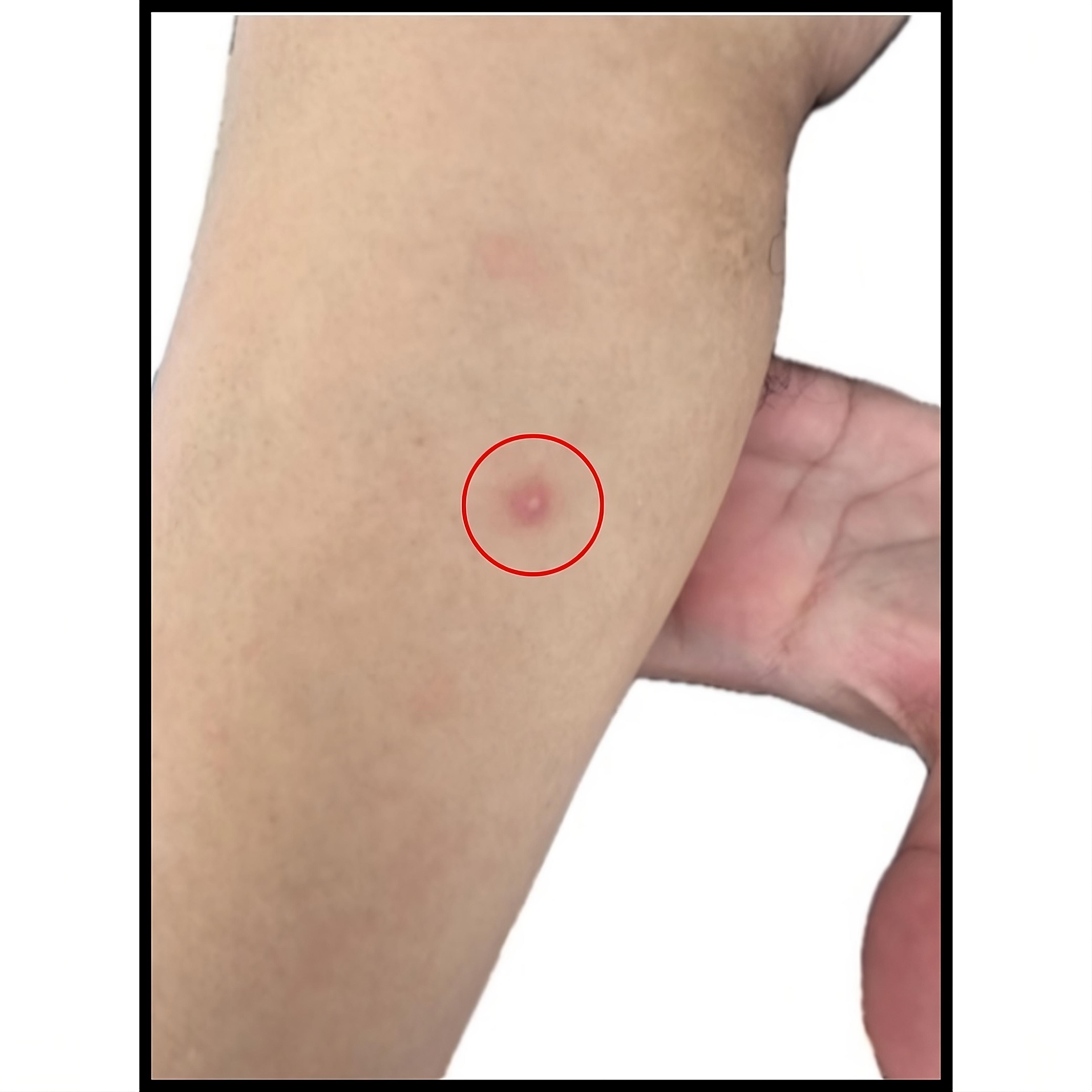

Supplement: Supplementary file 1 [file vaccines-13-00210-s001.zip › S3B.jpg]
